# A systematic review to describe patterns of animal and human viral research in Rwanda

**DOI:** 10.1093/inthealth/ihac031

**Published:** 2022-06-01

**Authors:** M Fausta Dutuze, Maurice Byukusenge, Anselme Shyaka, Rebecca C Christofferson

**Affiliations:** Rwanda Institute for Conservation Agriculture, Gashora, Bugesera, Rwanda; Animal Diagnostic Laboratory, Pennsylvania State University, University Park, PA 16802, USA; College of Agriculture and Animal Sciences and Veterinary Medicine, University of Rwanda, Kigali, Rwanda; Center for One Health, University of Global Health Equity, 23WV + R53, Kigali, Rwanda; Department of Pathobiological Sciences, School of Veterinary Medicine, Louisiana State University, Baton Rouge, LA 70803, USA

**Keywords:** One Health, policy, research, Rwanda, viral diseases, viruses

## Abstract

Rwanda is located in the Central East African region where several viral pathogens with global importance were originally described, including human immunodeficiency virus (HIV), Ebola, Zika, Rift Valley Fever (RVF), dengue and a long list of other neglected tropical viral pathogens. Due to many factors, this region has the potential to become a global hotspot for viral emergence. In Rwanda, viral diseases are underreported and the question is whether this is due to the absence of these viruses or a lack of investigation. Like many developing countries, capabilities in Rwanda need improvement despite research efforts throughout the years. This review describes the status of human and animal virus research in Rwanda and identifies relevant research and operational gaps. A comprehensive search was conducted in PubMed for virus research in Rwanda: 233 primary studies on viruses/viral diseases are indexed with connection to Rwanda. From 1958 to 2020, yearly publications generally increased and HIV/acquired immunodeficiency syndrome is the most studied virus. Compared with human viruses, few studies focus on animal and/or zoonotic viruses. The occurrence of the current severe acute respiratory syndrome coronavirus 2 pandemic shows strengthening warning and surveillance systems is critical to efficient preparedness and response. We recommend investment in human capacity, laboratory facilities and research to inform policy for viral surveillance in Rwanda.

## Introduction

Rwanda is located in the Central East African region, which has been identified as one of the major global hotspots of emerging infectious diseases.^1^ Many of the ecological and human factors contributing to the emergence (and re-emergence) of infectious diseases (EIDs)—including population growth, increased urbanization and human mobility—are prevalent in Rwanda.^2^ According to the World Health Organization (WHO), EIDs are among the biggest challenges of the 21st century, as they not only pose a threat to public and animal health, but also constitute a hindrance for socio-economic growth.^3^

Although EIDs include both bacterial and viral diseases, viral diseases are a majority proportion of the recently identified globally emerging diseases, and most outbreaks in the last 5 decades, including the current severe acute respiratory syndrome coronavirus 2 (SARS-CoV-2) pandemic, have been caused by viruses.^4,5^ Several viruses, including haemorrhagic fever viruses like Marburg, Ebola, chikungunya, dengue and O’nyong-nyong viruses, have been identified in the immediate neighbours of Rwanda, most notably Uganda,^6–8^ the Democratic Republic of Congo^9^ and Tanzania.^10,11^ As shown by the impact of the recent epidemics caused by such viruses in Central and West African countries, the vulnerability of the world to viral epidemics is mainly due to the lack of preparedness and weak surveillance systems.^12,13^

One of the lessons learned amid the current SARS-CoV-2 pandemic is that effective disease prevention requires the availability of strong systems for early warning of pathogens with epidemic potential. Risk assessment, proper communication channels between different response entities and laboratory capacity are some of the key areas of operational response and preparedness that are crucial to effective prevention and control of EIDs. Critical to operational success is infrastructure. Recently, remarkable efforts have been invested in building laboratory infrastructure, with reported preliminary success.^14,15^ Despite those efforts, Rwanda, like many other developing countries, is still lagging in some of the key areas of preparedness for EID epidemics. For example, as highlighted in the recently developed Rwanda One Health policy,^16^ human resource capacity in the laboratories is still deficient. As in many African countries, there are many challenges, including geographical barriers, cost and human resource pipelines.^17^ Importantly, communication channels between entities involved in surveillance and prevention of animal and human diseases need to be reinforced, since most emerging and re-emerging viral diseases are zoonotic in nature and their risk factors are both human and environmental. Thus prevention and preparedness requires a One Health approach with investment in infrastructure and involvement of experts across various disciplines and professions.^18^

Critical to developing such a response is understanding the baseline landscape of what is known about viral diseases, which plays an important role in establishing optimized prevention and control programs. For example, understanding the transmission cycle of the pathogens as well as the pathogen–host interactions involved in pathogenesis and disease presentation are important for robust prevention and control strategies.^19^ Collating the existing prior knowledge as a baseline to understand the current status of human and animal viruses and viral diseases in a region is necessary to move forward with designing these strategies and associated responses. By reviewing the existing studies that make up the body of knowledge regarding viral diseases in Rwanda, we will determine the baseline landscape of viral diseases of One Health importance and identify challenges to research. Moreover, the review will identify which of the four aspects (diagnostics, epidemiology, infectious kinetics, therapeutics and prevention) of research in these viruses and viral diseases were given more importance and therefore elucidates where more efforts should be focused for more holistic research in this area. The overall goals of this study were to understand the landscape of virological research in Rwanda, to identify gaps in both virology and research needs, as well as to understand how infrastructure and resources are needed to bolster Rwandan research as well as Rwandan researchers.

## Methods

For the purposes of this review, throughout the article, ‘virus’ research indicates human and/or animal virus research, as that is the primary focus of this effort.

### Search strategy

We performed a primary search in PubMed for scientific literature related to human and animal virus studies in Rwanda. This online database was used to find articles published from its inception until 1 March 2022. The Medical Subject Headings (MeSH) search terms were ‘((Rwanda) AND (virus OR viral disease OR Poxviridae OR Asfarviridae OR Iridoviridae OR Herpesviradae OR Adenoviridae OR Papillomaviridae OR Polyomaviridae OR Parvoviridae OR Circoviridae OR Anelloviridae OR Retroviridae OR Reoviridae OR Birnaviridae OR Picobirnaviridae OR Paramyxoviridae OR Pneumoviridae OR Rhabdoviridae OR Filoviridae OR Bornaviridae OR Orthomyxoviridae OR Bunyaviridae OR Arenaviridae OR Coronaviridae OR Arteriviridae OR Roniviridae OR Picornaviridae OR Caliciviridae OR Astroviridae OR Togaviridae OR Flaviviridae OR Hepeviridae OR Hepadnaviridae OR Deltaviruses OR Nodaviridae))’. The keywords were constructed based on virus families reported in Fenner's Veterinary Virology.^20^

A secondary, targeted search was performed using the Europe PMC, which includes sources not always returned in PubMed alone. To do this, we performed three smaller searches with the following keywords: (Rwanda AND ‘animal viruses’), (Rwanda AND ‘human viruses’) and (Rwanda AND (Poxviridae OR Asfarviridae OR Iridoviridae OR Herpesvirales OR Adenoviridae OR Papillomaviridae OR Polyomaviridae OR Parvoviridae OR Circoviridae OR Anelloviridae OR Retroviridae OR Reoviridae OR Birnaviridae OR Picobirnaviridae OR Paramyxoviridae OR Pneumoviridae OR Rhabdoviridae OR Filoviridae OR Bornaviridae OR Orthomyxoviridae OR Bunyaviridae OR Arenaviridae OR Coronaviridae OR Arteriviridae OR Roniviridae OR Picornaviridae OR Caliciviridae OR Astroviridae OR Togaviridae OR Flaviviridae OR Hepeviridae OR Hepadnaviridae OR Deltaviruses OR Nodaviridae).

### Inclusion and exclusion criteria

The inclusion criteria were articles presenting original research, observational studies and/or secondary data analysis. The obtained articles were screened for any of the following exclusion criteria: review articles, studies focusing exclusively on human societal aspects of viral diseases (e.g. studies on Knowledge Attitudes and Practice, mental health of patients, etc.), prevention studies that do not target the virus itself or virus-infected population, research on viruses that do not affect animal or human health and studies on populations outside Rwanda. The period included all studies in PubMed through 1 March 2022.

### Review of articles

**Figure 1.**
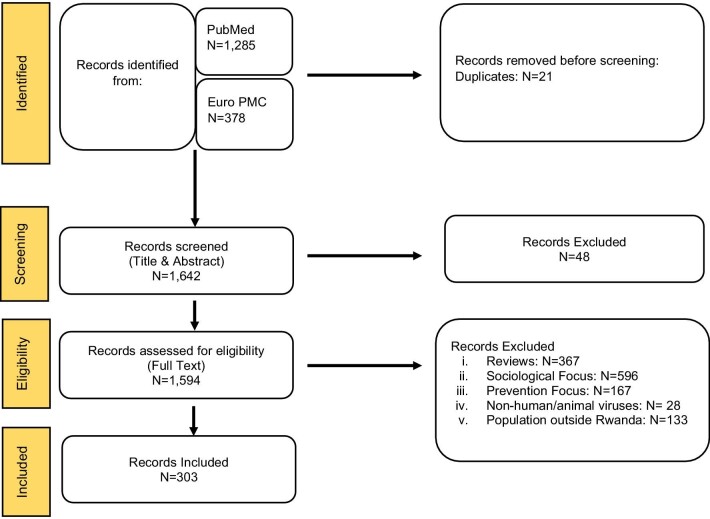
Schematic representing the systematic review of the available research for human or animal viruses and viral diseases in Rwanda.

All articles returned from the MeSH search were further screened for eligibility by checking the title and abstract to infer relevance. The original plan included that all articles would be screened by two or three individuals on the team and if there was a discrepancy, a discussion would be held to produce a consensus. However, in practice, there was a consensus in the initial screening phase. In case the abstract and title were not enough for an informed decision, the full article was evaluated for inclusion and exclusion criteria (Figure 1). Thus an article was confirmed eligible following a consensus of reviewers. A full list of references is provided in the supplementary materials.

The 303 articles included were then classified into four research themes: epidemiology, infection kinetics, diagnostics and therapeutics and prevention. The studies classified into the epidemiology category included those on prevalence, surveillance, risk factors and mathematical modelling. Infection kinetics studies were lab-based studies of infection dynamics *in vivo* and *in vitro*. Diagnostics studies were focused on the development of diagnostic tools, while studies in the therapeutics and prevention category focused on the development of therapeutics and prevention tools, including clinical trials. Further, studies were then evaluated according to the virus targeted by the research study, the primary vertebrate host of the virus investigated, the type of study and the involvement of Rwandan research institutions in the studies.

## Results

### History of published viral research in Rwanda

**Figure 2.**
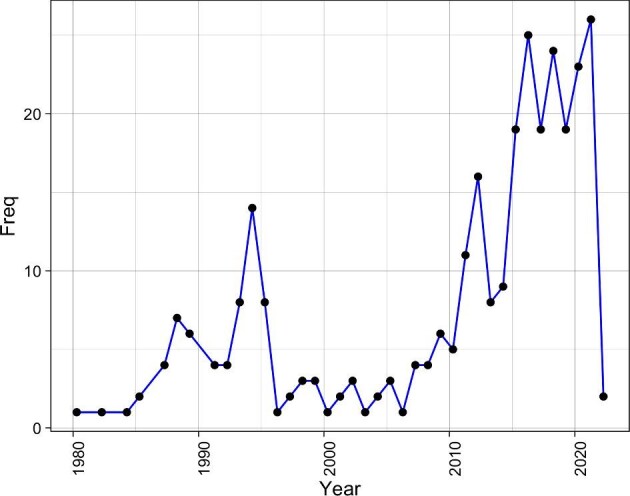
The number of publications on human or animal virus research in Rwanda from 1980 to 2020 (n=232) included in the 233 publication meeting inclusion criteria. The first publication from 1958 was not included in the viewing frame of this figure.

The first study to address a virus reported in Rwanda was a clinical trial of a vaccine against poliomyelitis published in 1958.^21^ Subsequently there was a 22-y gap between that article and the next indexed study published in 1980.^22^ This study reported on the prevalence of skin diseases, including those with viral aetiologies, and marked the beginning of a sustained trend of viral research in Rwanda. Since then, scientific articles on human or animal virus research have been published at a rate of at least one every 3 y, with a general increasing trend (Figure 2).

The overall trend of virus research in Rwanda is characterized by three phases: initial phase in the late 1980s to 2000, stagnant phase from 2000 to 2010 and a relatively exponential phase from 2010 onward (with the obvious exception of 2020 when a global shutdown paused much global research). The initial step in 1980 corresponds to the slow reorganization of the country after obtaining independence in 1962. As for many other sectors in Rwanda, virus research was stagnant in the post-genocide era against the Tutsi in 1994. This is illustrated by the stagnant phase in 2000–2010, as the country was rebuilding from the damage caused by this devastating historical event. In 2010, there appears to be a relaunch of virus research in Rwanda, which has been showing a promising ascending curve since (Figure 2).

Table 1 shows the breakdown of the studies by themes and identified patterns. The majority of virus research studies in Rwanda are on epidemiology (65.6% [199/303]). Therapeutics and prevention studies represent the second most frequent type of study (21.1% [64/303]). The least represented research themes are diagnostics and infection kinetics studies, which represent 13.2% (40/303) of all reported studies (Table 1).

**Table 1.** Number of research articles per research category and research theme

	Research themes [references]				
Main research categories	Diagnostics	Epidemiology	Infection kinetics	Therapeutics and prevention	Total
Studies on HIV only	13 [1–13]	92 [14–105]	22 [106–127]	43 [128–170]	170
Studies combining HIV and other viruses	0	15 [171–185]	0	1 [186]	16
Studies on non-HIV viruses only	4 [187–190]	92 [191–282]	1 [283]	20 [284–303]	117
Total	17	199	23	64	303

In each cell, the first number denotes the number of articles per category and per research theme. The reference numbers (in square brackets) indicate the numbers in the bibliography in the supplementary materials.

Human immunodeficiency virus (HIV) research generally dominated all virus-related studies, with 56.1% (170/303) of all reported investigations. This high proportion reflects the significant interest in this virus with both pandemic potential and high lethality that was emerging in Rwanda and neighbouring countries in the 1980s. Subsequently, this considerable interest in HIV marked the growth of virus research in Rwanda after a 22-y gap. Given its public health relevance, HIV still represents an important pillar in this field of study in Rwanda and the region overall.

### HIV research as a springboard for virus research in Rwanda

The number of research papers on HIV and other viruses in Rwanda published over the past 4 decades seems to indicate that research on viral diseases in Rwanda was kick-started by various kinds of investigations on HIV/acquired immunodeficiency syndrome (AIDS). As the world was becoming aware of AIDS and its causative virus, global organizations led by the World Health Organization (WHO) made investments aimed at preventing HIV/AIDS from becoming a global pandemic. At that time, Rwanda was at the epicentre of this devastating disease that is still without a widely available cure or vaccine. It is thus not surprising that Rwanda was among the countries that were the target of research projects aimed at better understanding this virus, its risk factors and its transmission dynamics. The country's institutional capacity was, at best, in its infancy, as the country was beginning to establish its economy in the wake of the colonial era.^23^

**Figure 3.**
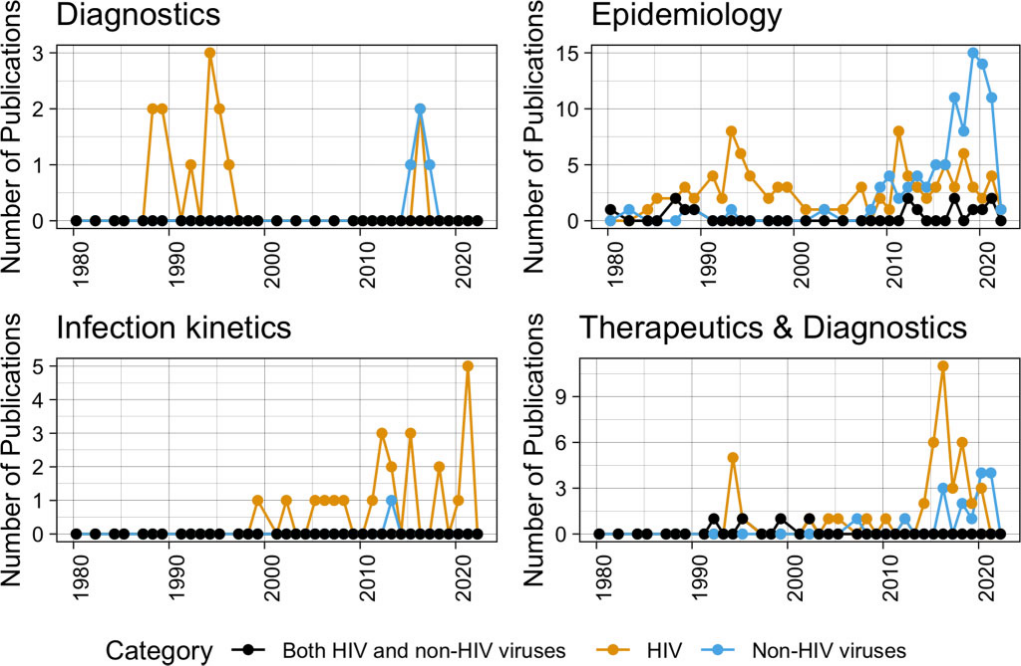
Comparative trends of yearly numbers of publications focused exclusively on HIV, those focused exclusively on non-HIV human or animal viruses or publications including both HIV and non-HIV animal or human viruses.

The annual number of research papers on HIV in Rwanda increased at a slow rate and reached its highest level in the mid-1990s and suddenly decreased again to the previous minimum effort, which coincided with the genocide against the Tutsi in 1994. This had obvious repercussions for institutional capacity in all areas of development, including training of researchers capable of conducting any research on diseases. In the early 2010s, the country's efforts to rebuild its institutions were being realized and research on viruses started to increase again, not only for HIV, but also for other viruses (Figure 3).

### Non-HIV human viruses of interest

This literature review identified a total of 98 articles reporting non-HIV viral research pertaining to human infection. In these 98 articles, a total of 95 viruses were identified, and the most frequently identified viruses were hepatitis B, hepatitis C, human papillomavirus (HPV), rotavirus, measles virus and herpesvirus. These six viruses accounted for 77.1% of the publications on non-HIV viruses (76/98). Epidemiological studies were dominant in the 98 articles and represented 76.5% (75/98), while therapeutics and prevention, diagnostics and infection kinetics accounted for 18.3% (18/98), 4.1% (4/98) and 0.1% (1/98), respectively. The first non-HIV viral research was published in 1957; however, the biggest proportion of the non-HIV studies (77.5% [76/98]) was published in the last 5 y (2015–2020). Most of these 76 studies are of an epidemiological nature and consist mainly of establishing the prevalence of the viruses in various risk groups.

Rwanda has been hailed for pioneering the introductions of various vaccines, including the vaccines against hepatitis B, HPV and rotavirus. This move was aimed at meeting Millennium Development Goal 4 (reduce mortality in children <5 y of age by two-thirds by 2015).^24^ Data from the Demographic and Health Survey of 2010 showed that mortality in the <5-year-old age group dropped from 103 to 76 deaths per 1000 between 2008 and 2010, indicating that some success was achieved. We argue that this push to eradicate the above major diseases, associated with mass vaccinations and screening of children or at-risk populations, may explain the dominance and relatively recent increase in the publication of scientific reports on these non-HIV viruses. However, this speaks to a reactive basic research paradigm rather than a proactive one. That is, basic research appears to follow the clinical research programs rather than the other way around. While this is appropriate for pharmaceutical research and development, basic research is still necessary to inform preparedness and responses for EIDs.

### Virus research in non-human animal hosts

This review identified only 21 viral studies on animals (domestic and wildlife). These research reports were of an epidemiological nature and of the 21 articles, 14 were carried out on non-human primates, accounting for 100% of the wildlife studies.^25–35^ In contrast, there was a clear scarcity of data (seven studies) on virus research in domestic animals,^36,37^ both of which were led by researchers affiliated with foreign institutions, suggesting non-governmental funding was used to carry out these investigations.

The relative abundance of viral research in wildlife compared with domestic animals can likely be explained by various factors. Rwanda has a high interest in wildlife conservation for tourist purposes. In fact, Rwanda is one of very few countries that hosts the endangered mountain gorilla (*Gorilla beringei beringei*). This country's interest has attracted various projects that aim at contributing to the protection of endangered species and wildlife in general. Among these projects, Gorilla Doctors are dedicated to protecting mountain and eastern lowland gorillas through provision of veterinary care.^38^ In addition, PREDICT, a United States Agency for International Development–funded project, working closely with Gorilla Doctors, has led most research initiatives on non-human primates and other wildlife.^39^ The financial capability of these two projects, in addition to their affiliations with established and experienced overseas research institutions, can partially explain the dominance of research in wildlife compared with other animals in the country.

### Zoonotic aspect in virus research: are we there yet?

Despite the threat posed by zoonotic diseases in the central African region, only 12.5% (38/303) of studies of viruses in Rwanda focused on zoonoses. Four of these were published prior to 2010, with one or more articles published per year after 2011. All these studies focused on the epidemiological aspects of these viruses/diseases. Twenty-two (57.8%) were about the epidemiology of viral diseases in human populations. In most of them, the zoonotic characteristics (transmission cycle, reservoir, etc.) of these viruses were not a major focus of the investigations. An additional 14 studies focused on the epidemiology of viruses in animals, and most of the zoonotic viruses investigated in animals were from the African great apes due to their economic and ecological importance.^25,26,29,30^,^32–34^ Only three studies^31^ investigated the potential for cross-transmission adenoviruses between humans and animals (two on human adenoviruses and one on circoviruses).

Of the four articles identified in this review that had a clear intent of investigating the zoonotic potential, two^27,35^ investigated the presence of paramyxoviruses and coronaviruses in bats and the other two^36,37^ investigated the presence of viruses of the Bunyamwera family in cattle. Regardless of whether zoonotic potential was a focus of the article, the zoonotic viruses most studied in humans were the influenza viruses and other respiratory viruses, while coronaviruses, paramyxoviruses, Bunyaviruses, various respiratory viruses and simian retroviruses were the most investigated in animals.

### Thematic evolution for virus research: from prevalence to molecular studies

**Figure 4.**
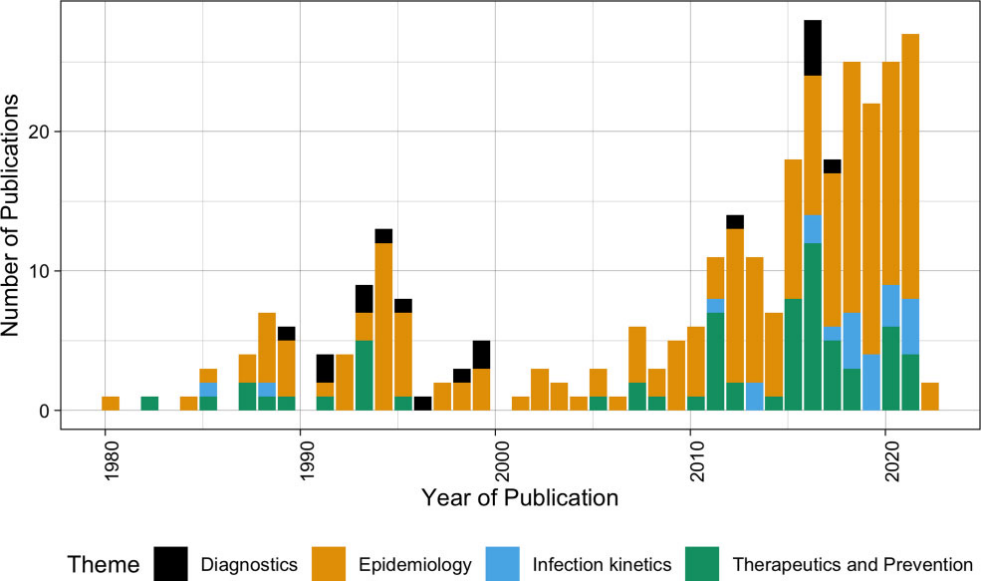
Thematic evolution of human or animal virus research in Rwanda.

The studies on epidemiological aspects of viruses/viral diseases were the earliest to be reported^22^,^40–50^ and constitute the most frequently published type of study on viruses in Rwanda (Figure 4 and Table 1). Studies in this research theme include mainly screenings using conventional serological and simple molecular laboratory assays and/or questionnaire-based surveys investigating risk factors. This type of research does not often require highly sophisticated laboratory equipment and thus are the easiest to conduct in less developed research conditions.

**Figure 5.**
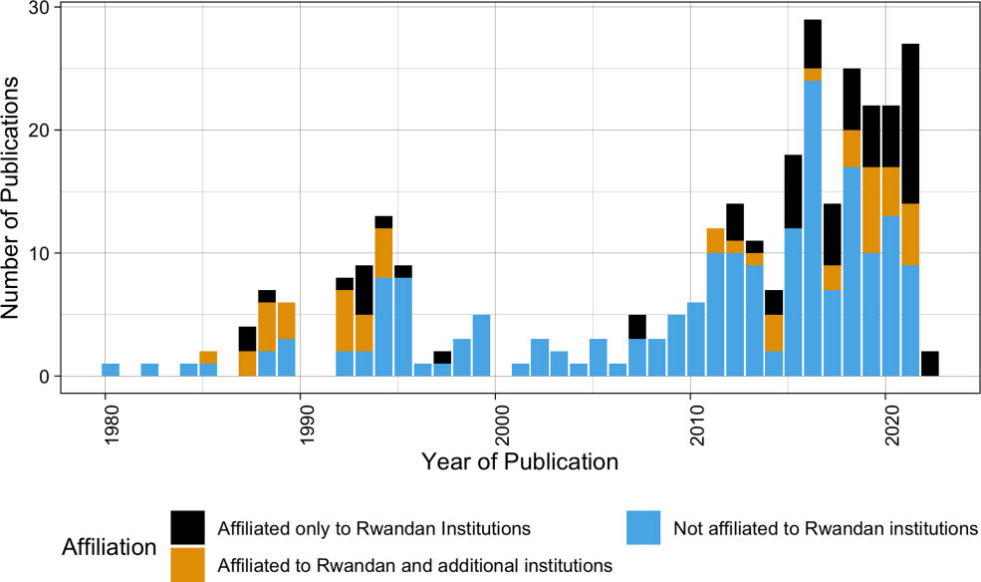
Affiliation of first authors of publications on human or animal virus research in Rwanda.

Investigations into the development of laboratory assays for detection of viruses or markers of infection, which were classified into the diagnostics research theme, were also among the earliest to be conducted, with the earliest focused on the development of HIV/AIDS diagnostic tools in the late 1980s.^51–54^ HIV was the subject of most diagnostic studies, where 15/17 studies focused on HIV (Table 1). The remaining two studies focused on diagnostic tools of canine distemper and Epstein–Barr virus.^55,56^

The infection kinetics research theme was not represented before 1999^57^ and has continued at a low rate compared with other types of research. This is likely because lab capacity (such as high-containment laboratories [biosafety level 3 or 4]), reliable cold chain systems and access to and/or appropriate maintenance of advanced molecular technologies are not consistently available to support projects in Rwanda. Thus this type of research has been limited within Rwanda and relies on research partners outside the country. Again, all infection kinetics themed studies have focused on HIV, which highlights its significant representation (Table 1).

### Role of Rwandan institutions in viral research in Rwanda

We evaluated the role of Rwandan researchers by identifying the first authors and determining whether that author had an affiliation with a Rwandan institution. First authors are often either graduate students or other research leads (sometimes under the direction of senior authors), so we focused on this as an indication of lead authorship. The beginning of the growth of viral research in Rwanda was led by non-Rwandan institutions in early 1980s.^22,40,41^ In the mid-1980s, Rwandan scientists and institutions began to be represented through authorship, often with double affiliations and conducting research in conjunction with or at institutions outside of Rwanda. Unfortunately, this positive trend was interrupted by the 1994 genocide, which caused a long-term gap in many developmental trends in Rwanda (Figure 5). In the subsequent 15 y, the few viral research projects in Rwanda were conducted by first authors not affiliated with Rwandan institutions (Figure 4). From 2010, Rwandan institutions began to be more represented as leads in viral research conducted in the country. This is characterized by an increasing number of first authors affiliated with Rwandan institutions, whether exclusively or with dual affiliations. However, since 2010, first authors have often been affiliated with western institutions.

Overall, only 37.9% (115/303) of publications reporting on virus research in Rwanda have first authors affiliated with Rwandan institutions. Fifty-six of these were solely affiliated with Rwandan institutions, while 49 others were affiliated with both a Rwandan and a foreign institution. First authors affiliated with foreign institutions—whether uniquely or additionally to Rwandan institutions—were mainly affiliated with institutions in North America and Europe: United States (30), Belgium (24), Sweden (11), United Kingdom (5), Luxembourg (2), Germany (2), France (1), Greece (1) and Canada (1). A few authors were affiliated with institutions in Asia and other African countries: China (4), South Africa (3), Kenya (2), Nigeria (1), India (1) and Taiwan (1).

Although significant progress has been made towards appropriate inclusion of Rwandan scientists and institutions in Rwandan research, the proportion of Rwanda institutions that independently take the lead in virus research remains limited. The limited number of Rwandan experts in the field of virology who remain in Rwanda, less advanced laboratory equipment in research and higher learning institutions, underdeveloped virology-related programs in institutions of higher learning and limited national funding programs to support such research are among the factors that likely contribute to this trend.

Of the 303 publications considered in this study, 263 were lab-based. The remaining are questionnaire-based studies. However, in these 263 lab-based studies, in only a few was the laboratory work conducted in Rwanda. Only 38.7% (102/263) were entirely conducted in Rwanda, while 39.9% (121/303) were entirely performed outside Rwanda, with the remaining studies (40/303) having a hybrid approach where the preliminary analyses were conducted in Rwanda with further sophisticated laboratory analyses performed abroad. A majority (61.2% [87/142]) of studies with laboratory analyses either entirely or partially conducted in Rwanda focused on HIV.

### Strengths and limitations of the systematic review

This review is intended to describe the state of research pertaining to animal and human viruses in Rwanda and represents an important step in understanding the landscape of research in order to identify and prioritize needs. However, it is not without limitations. There is always the possibility that some research is ‘missed’ in internet searches, although we believe our search was thorough. Further, we do not address plant viruses, which constitutes and important aspect of research and One Health. We encourage such review from experts in that field in order to really understand the entirety of virus research in the country.

### Is the current state of virus research in Rwanda enough to inform virus prevention and control policies?

Previous studies have highlighted the importance of research-based policy formulations in developed and developing countries.^58–60^ In fact, the last decades have been characterized by an improvement in human well-being marked by a reduction in child mortality and increased life expectancy in Rwanda. The research community—both Rwandan and international—have contributed to this enhanced well-being through contributions to science and health outcomes research. However, translation of research overall into policy is still a challenge.^59^ In order for Rwandan viral research to continue its upward trend and support not only Rwandan communities, but Rwandan scientists and institutions, the following three sectors should be better supported.

#### Human capacity

The availability of sufficiently trained staff at all levels of scientific investigation is needed to lead and conduct viral research in Rwanda. Human capacity must be improved to support Rwandan scientists who are willing and able to take a leading role in the research process, including grant writing, data collection, analyses and results dissemination. The findings from this review highlight a shortage of involvement of Rwandan institutions in published articles and the use of laboratories located overseas for Rwandan-relevant research. These two findings may be due, at least in part, to the lack of a sufficient workforce available to adequately staff local laboratories and research institutions.

Furthermore, the increasing research on viruses in Rwanda, as in many other African countries, emanates from the research communities’ interest in investigating the epidemiological aspects of viruses that may be specific to different geographic environments. Also, increasing the number of Rwandan researchers makes it easier to attract international collaborators who can add value to teams that must include Rwandan stakeholders.

#### Laboratory facilities

Research studies on HIV have undoubtedly contributed to the past capacity building of laboratory facilities, which can serve as a foundation for future infrastructure improvement endeavours. In contrast, little further development has been accomplished in recent years, and existing infrastructure may be skewed to the human side rather than veterinary services, as shown by the paucity of information available on domestic and wildlife viral studies. In the wake of emerging and re-emerging viruses with zoonotic potential, and the economic importance of livestock in Rwanda, it is important to simultaneously strengthen veterinary services for surveillance and enhance diagnostic capabilities. Thus equal focus should be given to human and animal lab capacities, with a focus on a One Health approach.

#### Investment in research to inform policy

Across most sectors in Rwanda there has been considerable positive growth in the period post-genocide. However, there has been comparatively modest increases in research in Rwanda, and the capacity for Rwandans to lead Rwanda science remains tenuous. Similar research-oriented investments must be aligned with the goal of utilizing local talent to tackle local issues in order to remain relevant and create a sustainable research ecosystem. Further, the increased investment means not only availing more sources of funds to carry out research relevant to Rwanda and the region, but also to define research priorities and directions that are inherently relevant to the Rwandan people. Research aspects such as the identification of risk factors for the occurrence of various viruses, modelling disease dynamics and host/vector distributions as well as studies on viral virulence and molecular characteristics should be prioritized in Rwanda.

### Conclusions

This review highlighted four main findings. First, most of the viral research in Rwanda has been focused on HIV, with relatively minor involvement of Rwandan institutions. Other virus research was a secondary objective of HIV research or was consistently published only in very recent years (after 2010) as scientific interest and capacity has evolved. Second, the totality of the published research regarding viruses and viral diseases of animals and humans in Rwanda mainly provide a basic baseline that lacks in-depth analyses or follow-up, such as determining definitive aetiologies, investigating pathogenesis in Rwandan populations and providing solution-based surveillance studies. Third, virus research in domestic animals and wildlife is relatively lacking, and the few available reports were mainly part of external funding related to the conservation of non-human primates rather than to the needed One Health paradigm to improve the health of Rwandans. Fourth, although viruses with known or suspected zoonotic potential were reported, only two studies were explicitly designed to evaluate the zoonotic nature of the investigated viruses. The three major sectors in need of bolstering are human capacity, laboratory facilities and investment in research to inform policy. Taken together, the results from this review suggest that Rwanda and the international community need to invest in and expand existing research through investment in capacity building in Rwanda in order to provide valuable information regarding viral diseases of humans and animals with global One Health importance.

## Supplementary Material

ihac031_Supplemental_File

## Data Availability

The data underlying this article will be shared upon reasonable request to the corresponding author.
