# Supplementary material for: A systematic review to describe patterns of animal and human viral research in Rwanda
Source: Int Health. 2022 Jun 1;15(2):113–22. doi: 10.1093/inthealth/ihac031 (PMC9384174; doi:10.1093/inthealth/ihac031)
Supplement: ihac031_Supplemental_File [file ihac031_supplemental_file.docx]

**A systematic review to describe patterns of animal and human viral research in Rwanda.**

**References**

1. Van de Perre P, Nzaramba D, Allen S *et al.* Comparison of six serological assays for human immunodeficiency virus antibody detection in developing countries. *J Clin Microbiol* 1988;26:552–6.

2. Van de Perre P, De Clercq A, Cogniaux-Leclerc J *et al.* Detection of HIV p17 antigen in lymphocytes but not epithelial cells from cervicovaginal secretions of women seropositive for HIV: implications for heterosexual transmission of the virus. *Genitourin Med* 1988;64:30–3.

3. Van de Perre P, Lepage P, Batungwanayo J *et al.* Adult and paediatric AIDS patients lacking HIV antibodies detected by enzyme immunoassay in central Africa: further serological studies. *AIDS* 1989;3:429–31.

4. Lepage P, Batungwanayo J, Van de Perre P. Seronegativity and HIV infection. *Arch Dis Child* 1989;64:135–7.

5. Van de Peere P, Lepage P, Simonon A *et al.* Biological markers associated with prolonged survival in African children maternally infected by the human immunodeficiency virus type 1. *AIDS Res Hum Retroviruses* 1992;8:435–42.

6. Simonon A, Lepage P, Karita E *et al.* An assessment of the timing of mother-to-child transmission of human immunodeficiency virus type 1 by means of polymerase chain reaction. *J Acquir Immune Defic Syndr (1988)* 1994;7:952–7.

7. Kerlikowske KM, Katz MH, Allen S *et al.* Beta 2-microglobulin as a predictor of death in HIV-infected women from Kigali, Rwanda. *AIDS* 1994;8:963–9.

8. Harms G, Kleinfeldt V, Bugingo G *et al.* Recognition of AIDS by health personnel in rural south-Rwanda. *Trop Med Parasitol* 1994;45:36–8.

9. Bulterys M, Farzadegan H, Chao A *et al.* Diagnostic utility of immune-complex-dissociated p24 antigen detection in perinatally acquired HIV-1 infection in Rwanda. *J Acquir Immune Defic Syndr Hum Retrovirol* 1995;10:186–91.

10. Fraisier C, Van de Perre P, Lepage P *et al.* Broadly neutralizing, MN-like PND-directed antibodies in Rwandan children with long-term HIV1 infection. *Res Virol* 1995;146:201–10.

11. Weber J, Fenyö EM, Beddows S *et al.* Neutralization serotypes of human immunodeficiency virus type 1 field isolates are not predicted by genetic subtype. The WHO Network for HIV Isolation and Characterization. *J Virol* 1996;70:7827–32.

12. Bergin PJ, Langat R, Omosa-Manyonyi G *et al.* Assessment of Anti-HIV-1 Antibodies in Oral and Nasal Compartments of Volunteers From 3 Different Populations. *J Acquir Immune Defic Syndr* 2016;73:130–7.

13. Landais E, Huang X, Havenar-Daughton C *et al.* Broadly Neutralizing Antibody Responses in a Large Longitudinal Sub-Saharan HIV Primary Infection Cohort. *PLoS Pathog* 2016;12:e1005369.

14. Van de Perre P, Rouvroy D, Lepage P *et al.* Acquired immunodeficiency syndrome in Rwanda. *Lancet* 1984;2:62–5.

15. Clumeck N, Robert-Guroff M, Van de Perre P *et al.* Seroepidemiological studies of HTLV-III antibody prevalence among selected groups of heterosexual Africans. *JAMA* 1985;254:2599–602.

16. Van de Perre P, Clumeck N, Carael M *et al.* Female prostitutes: a risk group for infection with human T-cell lymphotropic virus type III. *Lancet* 1985;2:524–7.

17. Van de Perre P, Le Polain B, Carael M *et al.* HIV antibodies in a remote rural area in Rwanda, Central Africa: an analysis of potential risk factors for HIV seropositivity. *AIDS* 1987;1:213–5.

18. Van de Perre P, Carael M, Nzaramba D *et al.* Risk factors for HIV seropositivity in selected urban-based Rwandese adults. *AIDS* 1987;1:207–11.

19. Van de Perre P, Hitimana DG, Lepage P. Human immunodeficiency virus antibodies of IgG, IgA, and IgM subclasses in milk of seropositive mothers. *J Pediatr* 1988;113:1039–41.

20. Carael M, Van de Perre PH, Lepage PH *et al.* Human immunodeficiency virus transmission among heterosexual couples in Central Africa. *AIDS* 1988;2:201–5.

21. Dunn D. Nationwide community-based serological survey of HIV-1 and other human retrovirus infections in a Central African country. *WHO AIDS Tech Bull* 1989;2:143–4.

22. Feldman DA. A household survey for AIDS-related complex in Rwanda. *Med Anthropol* 1989;10:143–9.

23. Lepage P, Van de Perre P, Van Vliet G *et al.* Clinical and endocrinologic manifestations in perinatally human immunodeficiency virus type 1--Infected children aged 5 years or older. *Am J Dis Child* 1991;145:1248–51.

24. Allen S, Lindan C, Serufilira A *et al.* Human immunodeficiency virus infection in urban Rwanda. Demographic and behavioral correlates in a representative sample of childbearing women. *JAMA* 1991;266:1657–63.

25. Van de Perre P, Simonon A, Msellati P *et al.* Postnatal transmission of human immunodeficiency virus type 1 from mother to infant. A prospective cohort study in Kigali, Rwanda. *N Engl J Med* 1991;325:593–8.

26. Lepage P, Dabis F, Hitimana DG *et al.* Perinatal transmission of HIV-1: lack of impact of maternal HIV infection on characteristics of livebirths and on neonatal mortality in Kigali, Rwanda. *AIDS* 1991;5:295–300.

27. Commenges D, Alioum A, Lepage P *et al.* Estimating the incubation period of paediatric AIDS in Rwanda. *AIDS* 1992;6:1515–20.

28. Lepage P, Van de Perre P, Simonon A *et al.* Transient seroreversion in children born to human immunodeficiency virus 1-infected mothers. *Pediatr Infect Dis J* 1992;11:892–4.

29. Bulterys M, Chao A, Dushimimana A *et al.* Multiple sexual partners and mother-to-child transmission of HIV-1. *AIDS* 1993;7:1639–45.

30. Bulterys M, Nzabihimana E, Chao A *et al.* Long-term survival among HIV-1-infected prostitutes. *AIDS* 1993;7:1269.

31. Karita E, Martinez W, Van de Perre P *et al.* HIV infection among STD patients--Kigali, Rwanda, 1988 to 1991. *Int J STD AIDS* 1993;4:211–3.

32. Moore PS, Allen S, Sowell AL *et al.* Role of nutritional status and weight loss in HIV seroconversion among Rwandan women. *J Acquir Immune Defic Syndr (1988)* 1993;6:611–6.

33. Van de Perre P, Simonon A, Hitimana DG *et al.* Infective and anti-infective properties of breastmilk from HIV-1-infected women. *Lancet* 1993;341:914–8.

34. Kurawige JB, Gatsinzi T, Kleinfeldt V *et al.* HIV-1 infection among malnourished children in Butare, Rwanda. *J Trop Pediatr* 1993;39:93–6.

35. Lepage P, Van de Perre P, Msellati P *et al.* Mother-to-child transmission of human immunodeficiency virus type 1 (HIV-1) and its determinants: a cohort study in Kigali, Rwanda. *Am J Epidemiol* 1993;137:589–99.

36. Bucyendore A, Van de Perre P, Karita E *et al.* Estimating the seroincidence of HIV-1 in the general adult population in Kigali, Rwanda. *AIDS* 1993;7:275–7.

37. Rübsamen-Waigmann H, von Briesen H, Holmes H *et al.* Standard conditions of virus isolation reveal biological variability of HIV type 1 in different regions of the world. WHO Network for HIV Isolation and Characterization. *AIDS Res Hum Retroviruses* 1994;10:1401–8.

38. De Wolf F, Hogervorst E, Goudsmit J *et al.* Syncytium-inducing and non-syncytium-inducing capacity of human immunodeficiency virus type 1 subtypes other than B: phenotypic and genotypic characteristics. WHO Network for HIV Isolation and Characterization. *AIDS Res Hum Retroviruses* 1994;10:1387–400.

39. Bulterys M, Chao A, Habimana P *et al.* Incident HIV-1 infection in a cohort of young women in Butare, Rwanda. *AIDS* 1994;8:1585–91.

40. Leroy V, Van de Perre P, Lepage P *et al.* Seroincidence of HIV-1 infection in African women of reproductive age: a prospective cohort study in Kigali, Rwanda, 1988-1992. *AIDS* 1994;8:983–6.

41. Chao A, Bulterys M, Musanganire F *et al.* Risk factors associated with prevalent HIV-1 infection among pregnant women in Rwanda. National University of Rwanda-Johns Hopkins University AIDS Research Team. *Int J Epidemiol* 1994;23:371–80.

42. Leroy V, Ntawiniga P, Nziyumvira A *et al.* HIV prevalence among pregnant women in Kigali, Rwanda. *Lancet* 1995;346:1488–9.

43. Leroy V, Msellati P, Lepage P *et al.* Four years of natural history of HIV-1 infection in african women: a prospective cohort study in Kigali (Rwanda), 1988-1993. *J Acquir Immune Defic Syndr Hum Retrovirol* 1995;9:415–21.

44. Lifson AR, Allen S, Wolf W *et al.* Classification of HIV infection and disease in women from Rwanda. Evaluation of the World Health Organization HIV staging system and recommended modifications. *Ann Intern Med* 1995;122:262–70.

45. Bulterys M, Chao A, Dushimimana A *et al.* HIV-1 seroconversion after 20 months of age in a cohort of breastfed children born to HIV-1-infected women in Rwanda. *AIDS* 1995;9:93–4.

46. Simonon A, Mulder-Kampinga GA, van de Perre P *et al.* Evolution of human immunodeficiency virus subtype A in women seroconverting post partum and in their offspring post-natally infected by ingestion of breast milk. *J Gen Virol* 1997;78 ( Pt 9):2225–33.

47. Kampinga GA, Simonon A, Van de Perre P *et al.* Primary infections with HIV-1 of women and their offspring in Rwanda: findings of heterogeneity at seroconversion, coinfection, and recombinants of HIV-1 subtypes A and C. *Virology* 1997;227:63–76.

48. Leroy V, Newell ML, Dabis F *et al.* International multicentre pooled analysis of late postnatal mother-to-child transmission of HIV-1 infection. Ghent International Working Group on Mother-to-Child Transmission of HIV. *Lancet* 1998;352:597–600.

49. Camp WL, Allen S, Alvarez JO *et al.* Serum retinol and HIV-1 RNA viral load in rapid and slow progressors. *J Acquir Immune Defic Syndr Hum Retrovirol* 1998;18:21–6.

50. Weng S, Bulterys M, Chao A *et al.* Perinatal human immunodeficiency virus-1 transmission and intrauterine growth: a cohort study in Butare, Rwanda. *Pediatrics* 1998;102:e24.

51. Tang J, Rivers C, Karita E *et al.* Allelic variants of human beta-chemokine receptor 5 (CCR5) promoter: evolutionary relationships and predictable associations with HIV-1 disease progression. *Genes Immun* 1999;1:20–7.

52. Tranchat C, Van de Perre P, Simonon-Sorel A *et al.* Maternal humoral factors associated with perinatal human immunodeficiency virus type-1 transmission in a cohort from Kigali, Rwanda, 1988-1994. *J Infect* 1999;39:213–20.

53. Spira R, Lepage P, Msellati P *et al.* Natural history of human immunodeficiency virus type 1 infection in children: a five-year prospective study in Rwanda. Mother-to-Child HIV-1 Transmission Study Group. *Pediatrics* 1999;104:e56.

54. Vangroenweghe D. The earliest cases of human immunodeficiency virus type 1 group M in Congo-Kinshasa, Rwanda and Burundi and the origin of acquired immune deficiency syndrome. *Philos Trans R Soc Lond B Biol Sci* 2001;356:923–5.

55. Salihu HM, Nnedu ON, Karita E *et al.* Predictors of HIV seropositivity following intrapartum voluntary counseling and testing among Rwandan women. *J Obstet Gynaecol* 2003;23:632–6.

56. Modjarrad K, Zulu I, Karita E *et al.* Predictors of HIV serostatus among HIV discordant couples in Lusaka, Zambia and female antenatal clinic attendants in Kigali, Rwanda. *AIDS Res Hum Retroviruses* 2005;21:5–12.

57. Tumusiime DK, Musabeyezu E, Mutimurah E *et al.* Over-reported peripheral neuropathy symptoms in a cohort of HIV infected and uninfected Rwandan women: the need for validated locally appropriate questionnaires. *African Health Sciences* 2014;14:460.

58. Li X, Zhang K, Pajewski NM *et al.* Immunogenetic influences on acquisition of HIV-1 infection: consensus findings from two African cohorts point to an enhancer element in IL19 (1q32.2). *Genes Immun* 2015;16:213–20.

59. Kamali A, Price MA, Lakhi S *et al.* Creating an African HIV clinical research and prevention trials network: HIV prevalence, incidence and transmission. *PLoS One* 2015;10:e0116100.

60. Borgdorff H, Verwijs MC, Wit FWNM *et al.* The impact of hormonal contraception and pregnancy on sexually transmitted infections and on cervicovaginal microbiota in african sex workers. *Sex Transm Dis* 2015;42:143–52.

61. Bendavid E, Stauffer D, Remera E *et al.* Mortality along the continuum of HIV care in Rwanda: a model-based analysis. *BMC Infect Dis* 2016;16:728.

62. Bórquez A, Cori A, Pufall EL *et al.* The Incidence Patterns Model to Estimate the Distribution of New HIV Infections in Sub-Saharan Africa: Development and Validation of a Mathematical Model. *PLOS Medicine* 2016;13:e1002121.

63. Kayigamba FR, Van Santen D, Bakker MI *et al.* Does provider-initiated HIV testing and counselling lead to higher HIV testing rate and HIV case finding in Rwandan clinics? *BMC Infectious Diseases* 2016;16:26.

64. Nsanzimana S, Remera E, Kanters S *et al.* Household survey of HIV incidence in Rwanda: a national observational cohort study. *The Lancet HIV* 2017;4:e457–64.

65. Mutagoma M, Samuel MS, Kayitesi C *et al.* High HIV prevalence and associated risk factors among female sex workers in Rwanda. *Int J STD AIDS* 2017;28:1082–9.

66. Wall KM, Rida W, Haddad LB *et al.* Pregnancy and HIV Disease Progression in an Early Infection Cohort from Five African Countries. *Epidemiology* 2017;28:224–32.

67. Riedel DJ, Stafford KA, Memiah P *et al.* Patient-level outcomes and virologic suppression rates in HIV-infected patients receiving antiretroviral therapy in Rwanda. *Int J STD AIDS* 2018;29:861–72.

68. Aluisio AR, Garbern S, Wiskel T *et al.* Mortality outcomes based on ED qSOFA score and HIV status in a developing low income country. *Am J Emerg Med* 2018;36:2010–9.

69. Haas AD, Zaniewski E, Anderegg N *et al.* Retention and mortality on antiretroviral therapy in sub-Saharan Africa: collaborative analyses of HIV treatment programmes. *J Int AIDS Soc* 2018;21.

70. Abimpaye M, Kirk CM, Iyer HS *et al.* The impact of “Option B” on HIV transmission from mother to child in Rwanda: An interrupted time series analysis. *PLOS ONE* 2018;13:e0192910.

71. Dai L, Sweat MD, Gebregziabher M. Modeling excess zeros and heterogeneity in count data from a complex survey design with application to the demographic health survey in sub-Saharan Africa. *Stat Methods Med Res* 2018;27:208–20.

72. Mpunga T, Znaor A, Uwizeye FR *et al.* A case-control study of HIV infection and cancer in the era of antiretroviral therapy in Rwanda. *Int J Cancer* 2018;143:1348–55.

73. Price MA, Rida W, Kilembe W *et al.* Control of the HIV-1 Load Varies by Viral Subtype in a Large Cohort of African Adults With Incident HIV-1 Infection. *J Infect Dis* 2019;220:432–41.

74. Ntale RS, Rutayisire G, Mujyarugamba P *et al.* HIV seroprevalence, self-reported STIs and associated risk factors among men who have sex with men: a cross-sectional study in Rwanda, 2015. *Sex Transm Infect* 2019;95:71–4.

75. Ross J, Ribakare M, Remera E *et al.* High levels of viral load monitoring and viral suppression under Treat All in Rwanda - a cross-sectional study. *J Int AIDS Soc* 2020;23:e25543.

76. Veldhuijzen NJ, Braunstein SL, Vyankandondera J *et al.* The epidemiology of human papillomavirus infection in HIV-positive and HIV-negative high-risk women in Kigali, Rwanda. *BMC Infectious Diseases* 2011;11:333.

77. Veldhuijzen NJ, Vyankandondera J, van de Wijgert JH. HIV acquisition is associated with prior high-risk human papillomavirus infection among high-risk women in Rwanda. *AIDS* 2010;24:2289–92.

78. Singh DK, Anastos K, Hoover DR *et al.* Human Papillomavirus Infection and Cervical Cytology in HIV-Infected and HIV-Uninfected Rwandan Women. *J Infect Dis* 2009;199:1851.

79. Pirillo MF, Bassani L, Germinario EAP *et al.* Seroprevalence of hepatitis B and C viruses among HIV-infected pregnant women in Uganda and Rwanda. *J Med Virol* 2007;79:1797–801.

80. Murenzi G, Kanyabwisha F, Murangwa A *et al.* Twelve-Year Trend in the Prevalence of High-Risk Human Papillomavirus Infection Among Rwandan Women Living With HIV. *J Infect Dis* 2020;222:74–81.

81. Munymana JB, M’Kumbuzi VRP, Mapira HT *et al.* Prevalence of HIV among people with physical disabilities in Rwanda. *Central African Journal of Medicine* 2014;60:62–9.

82. Kemal KS, Anastos K, Weiser B *et al.* Molecular Epidemiology of HIV Type 1 Subtypes in Rwanda. *AIDS Res Hum Retroviruses* 2013;29:957–62.

83. Harbertson J, Grillo M, Zimulinda E *et al.* HIV seroprevalence, associated risk behavior, and alcohol use among male Rwanda Defense Forces military personnel. *AIDS Behav* 2013;17:1734–45.

84. Rusine J, Jurriaans S, Wijgert J van de *et al.* Molecular and Phylogeographic Analysis of Human Immuno-deficiency Virus Type 1 Strains Infecting Treatment-naive Patients from Kigali, Rwanda. *PLOS ONE* 2012;7:e42557.

85. Kilembe W, Keeling M, Karita E *et al.* Failure of a novel, rapid antigen and antibody combination test to detect antigen-positive HIV infection in African adults with early HIV infection. *PLoS One* 2012;7:e37154.

86. Manigart O, Boeras DI, Karita E *et al.* A gp41-based heteroduplex mobility assay provides rapid and accurate assessment of intrasubtype epidemiological linkage in HIV type 1 heterosexual transmission Pairs. *AIDS Res Hum Retroviruses* 2012;28:1745–55.

87. Ntaganira J, Hass LJ, Hosner S *et al.* Sexual risk behaviors among youth heads of household in Gikongoro, south province of Rwanda. *BMC Public Health* 2012;12:225.

88. Braunstein SL, Ingabire CM, Kestelyn E *et al.* High human immunodeficiency virus incidence in a cohort of Rwandan female sex workers. *Sex Transm Dis* 2011;38:385–94.

89. El-Sadr WM, Coburn BJ, Blower S. Modeling the impact on the HIV epidemic of treating discordant couples with antiretrovirals to prevent transmission. *AIDS* 2011;25:2295–9.

90. Braunstein SL, Ingabire CM, Geubbels E *et al.* High burden of prevalent and recently acquired HIV among female sex workers and female HIV voluntary testing center clients in Kigali, Rwanda. *PLoS One* 2011;6:e24321.

91. Karasi JC, Dziezuk F, Quennery L *et al.* High correlation between the Roche COBAS® AmpliPrep/COBAS® TaqMan® HIV-1, v2.0 and the Abbott m2000 RealTime HIV-1 assays for quantification of viral load in HIV-1 B and non-B subtypes. *J Clin Virol* 2011;52:181–6.

92. Muvunyi CM, Dhont N, Verhelst R *et al.* Evaluation of a new multiplex polymerase chain reaction assay STDFinder for the simultaneous detection of 7 sexually transmitted disease pathogens. *Diagn Microbiol Infect Dis* 2011;71:29–37.

93. Braunstein SL, Nash D, Kim AA *et al.* Dual Testing Algorithm of BED-CEIA and AxSYM Avidity Index Assays Performs Best in Identifying Recent HIV Infection in a Sample of Rwandan Sex Workers. *PLOS ONE* 2011;6:e18402.

94. Braunstein SL, Umulisa M-M, Veldhuijzen NJ *et al.* HIV diagnosis, linkage to HIV care, and HIV risk behaviors among newly diagnosed HIV-positive female sex workers in Kigali, Rwanda. *J Acquir Immune Defic Syndr* 2011;57:e70-76.

95. Chaillet P, Zachariah R, Harries K *et al.* Dried blood spots are a useful tool for quality assurance of rapid HIV testing in Kigali, Rwanda. *Trans R Soc Trop Med Hyg* 2009;103:634–7.

96. Peters PJ, Karita E, Kayitenkore K *et al.* HIV-infected Rwandan women have a high frequency of long-term survival. *AIDS* 2007;21 Suppl 6:S31-37.

97. Karita E, Price M, Hunter E *et al.* Investigating the utility of the HIV-1 BED capture enzyme immunoassay using cross-sectional and longitudinal seroconverter specimens from Africa. *AIDS* 2007;21:403–8.

98. Baalwa J, Wang S, Parrish NF *et al.* Molecular identification, cloning and characterization of transmitted/founder HIV-1 subtype A, D and A/D infectious molecular clones. *Virology* 2013;436:33–48.

99. Cheng-Mayer C, Homsy J, Evans LA *et al.* Identification of human immunodeficiency virus subtypes with distinct patterns of sensitivity to serum neutralization. *Proc Natl Acad Sci U S A* 1988;85:2815–9.

100. Arendt V, Amand M, Iserentant G *et al.* Predominance of the heterozygous CCR5 delta-24 deletion in African individuals resistant to HIV infection might be related to a defect in CCR5 addressing at the cell surface. *J Int AIDS Soc* 2019;22:e25384.

101. Lane C, Adair L, Bobrow E *et al.* Longitudinal interrelationship between HIV viral suppression, maternal weight change, breastfeeding, and length in HIV-exposed and uninfected infants participating in the Kabeho study in Kigali, Rwanda. *Ann Epidemiol* 2021;53:1-6.e1.

102. Remera E, Mugwaneza P, Chammartin F *et al.* Towards elimination of mother-to-child transmission of HIV in Rwanda: a nested case-control study of risk factors for transmission. *BMC Pregnancy Childbirth* 2021;21:339.

103. Umviligihozo G, Muok E, Nyirimihigo Gisa E *et al.* Increased Frequency of Inter-Subtype HIV-1 Recombinants Identified by Near Full-Length Virus Sequencing in Rwandan Acute Transmission Cohorts. *Front Microbiol* 2021;12:734929.

104. Rwibasira GN, Malamba SS, Musengimana G *et al.* Recent infections among individuals with a new HIV diagnosis in Rwanda, 2018-2020. *PLoS One* 2021;16:e0259708.

105. Nsanzimana S, Rwibasira GN, Malamba SS *et al.* HIV incidence and prevalence among adults aged 15-64 years in Rwanda: Results from the Rwanda Population-based HIV Impact Assessment (RPHIA) and District-level Modeling, 2019. *Int J Infect Dis* 2022;116:245–54.

106. Costello C, Tang J, Rivers C *et al.* HLA-B*5703 independently associated with slower HIV-1 disease progression in Rwandan women. *AIDS* 1999;13:1990–1.

107. Roman F, Karita E, Monnet A *et al.* Rare and new V3 loop variants in HIV-1-positive long-term non-progressors from Rwanda. *AIDS* 2002;16:1827–9.

108. Karita E, Price MA, Lakhi S *et al.* High Transmitter CD4+ T-Cell Count Shortly after the Time of Transmission in a Study of African Serodiscordant Couples. *PLOS ONE* 2015;10:e0134438.

109. Tang J, Li X, Price MA *et al.* CD4:CD8 lymphocyte ratio as a quantitative measure of immunologic health in HIV-1 infection: findings from an African cohort with prospective data. *Front Microbiol* 2015;6:670.

110. Yue L, Pfafferott KJ, Baalwa J *et al.* Transmitted Virus Fitness and Host T Cell Responses Collectively Define Divergent Infection Outcomes in Two HIV-1 Recipients. *PLOS Pathogens* 2015;11:e1004565.

111. Connolly S, Wall KM, Tang J *et al.* Fc-gamma receptor IIA and IIIA variants in two African cohorts: Lack of consistent impact on heterosexual HIV acquisition, viral control, and disease progression. *Virology* 2018;525:132–42.

112. Wiener HW, Shrestha S, Lu H *et al.* Immunogenetic factors in early immune control of human immunodeficiency virus type 1 (HIV-1) infection: Evaluation of HLA class I amino acid variants in two African populations. *Hum Immunol* 2018;79:166–71.

113. Amornkul PN, Karita E, Kamali A *et al.* Disease progression by infecting HIV-1 subtype in a seroconverter cohort in sub-Saharan Africa. *AIDS* 2013;27:2775–86.

114. Prentice HA, Porter TR, Price MA *et al.* HLA-B*57 versus HLA-B*81 in HIV-1 Infection: Slow and Steady Wins the Race? *Journal of Virology* 2013;87:4043–51.

115. Merino AM, Song W, He D *et al.* HLA-B signal peptide polymorphism influences the rate of HIV-1 acquisition but not viral load. *J Infect Dis* 2012;205:1797–805.

116. Masaisa F, Breman C, Gahutu JB *et al.* Ferroportin (SLC40A1) Q248H mutation is associated with lower circulating serum hepcidin levels in Rwandese HIV-positive women. *Ann Hematol* 2012;91:911–6.

117. Boeras DI, Hraber PT, Hurlston M *et al.* Role of donor genital tract HIV-1 diversity in the transmission bottleneck. *Proc Natl Acad Sci U S A* 2011;108:E1156-1163.

118. Baan E, de Ronde A, Luchters S *et al.* HIV type 1 mother-to-child transmission facilitated by distinctive glycosylation sites in the gp120 envelope glycoprotein. *AIDS Res Hum Retroviruses* 2012;28:715–24.

119. Spear GT, Zariffard MR, Chen HY *et al.* Positive association between HIV RNA and IL-6 in the genital tract of Rwandan women. *AIDS Res Hum Retroviruses* 2008;24:973–6.

120. Ndagije F, Baribwira C, Coulter JBS. Micronutrients and T-cell subsets: a comparison between HIV-infected and uninfected, severely malnourished Rwandan children. *Ann Trop Paediatr* 2007;27:269–75.

121. Lazaryan A, Lobashevsky E, Mulenga J *et al.* Human leukocyte antigen B58 supertype and human immunodeficiency virus type 1 infection in native Africans. *J Virol* 2006;80:6056–60.

122. Jiang Y, Karita E, Castor D *et al.* Characterization of CD8+ T lymphocytes in chronic HIV-1 subtype A infection in Rwandan women. *Cell Mol Biol (Noisy-le-grand)* 2005;51 Suppl:OL737-743.

123. Umviligihozo G, Cobarrubias KD, Chandrarathna S *et al.* Differential Vpu-Mediated CD4 and Tetherin Downregulation Functions among Major HIV-1 Group M Subtypes. *J Virol* 2020;94.

124. Wall KM, Karita E, Nyombayire J *et al.* Genital Abnormalities, Hormonal Contraception, and Human Immunodeficiency Virus Transmission Risk in Rwandan Serodifferent Couples. *J Infect Dis* 2021;224:81–91.

125. Hassan AS, Hare J, Gounder K *et al.* A Stronger Innate Immune Response During Hyperacute Human Immunodeficiency Virus Type 1 (HIV-1) Infection Is Associated With Acute Retroviral Syndrome. *Clin Infect Dis* 2021;73:832–41.

126. McInally S, Wall K, Yu T *et al.* Elevated levels of inflammatory plasma biomarkers are associated with risk of HIV infection. *Retrovirology* 2021;18:8.

127. Byiringiro FM, Manirakiza F, Ruhangaza D *et al.* Pathology Characteristics of Lymphomas in Rwanda: A Retrospective Study. *East Afr Health Res J* 2021;5:170–3.

128. Cheingsong-Popov R, Lister S, Callow D *et al.* Serotyping HIV type 1 by antibody binding to the V3 loop: relationship to viral genotype. WHO Network for HIV Isolation and Characterization. *AIDS Res Hum Retroviruses* 1994;10:1379–86.

129. Pau CP, Kai M, Holloman-Candal DL *et al.* Antigenic variation and serotyping of HIV type 1 from four World Health Organization-sponsored HIV vaccine sites. WHO Network for HIV Isolation and Characterization. *AIDS Res Hum Retroviruses* 1994;10:1369–77.

130. Gao F, Yue L, Craig S *et al.* Genetic variation of HIV type 1 in four World Health Organization-sponsored vaccine evaluation sites: generation of functional envelope (glycoprotein 160) clones representative of sequence subtypes A, B, C, and E. WHO Network for HIV Isolation and Characterization. *AIDS Res Hum Retroviruses* 1994;10:1359–68.

131. Bachmann MH, Delwart EL, Shpaer EG *et al.* Rapid genetic characterization of HIV type 1 strains from four World Health Organization-sponsored vaccine evaluation sites using a heteroduplex mobility assay. WHO Network for HIV Isolation and Characterization. *AIDS Res Hum Retroviruses* 1994;10:1345–53.

132. Osmanov S. HIV type 1 variation in World Health Organization-sponsored vaccine evaluation sites: genetic screening, sequence analysis, and preliminary biological characterization of selected viral strains. WHO Network for HIV Isolation and Characterization. *AIDS Res Hum Retroviruses* 1994;10:1327–43.

133. Sánchez-Palomino S, Dopazo J, Olivares I *et al.* Primary genetic characterization of HIV-1 isolates from WHO-sponsored vaccine evaluation sites by the RNase-A mismatch method. *Virus Res* 1995;39:251–9.

134. Cos P, Hermans N, De BT *et al.* Antiviral activity of Rwandan medicinal plants against human immunodeficiency virus type-1 (HIV-1). *Phytomedicine* 2002;9:62–8.

135. Servais J, Lambert C, Karita E *et al.* HIV type 1 pol gene diversity and archived nevirapine resistance mutation in pregnant women in Rwanda. *AIDS Res Hum Retroviruses* 2004;20:279–83.

136. Lemmer P, Schneider S, Schuman M *et al.* Determination of nevirapine and efavirenz in plasma using GC/MS in selected ion monitoring mode. *Ther Drug Monit* 2005;27:521–5.

137. Mutwa PR, Boer KR, Asiimwe-Kateera B *et al.* Safety and effectiveness of combination antiretroviral therapy during the first year of treatment in HIV-1 infected Rwandan children: a prospective study. *PLoS One* 2014;9:e111948.

138. Nsanzimana S, Remera E, Kanters S *et al.* Effect of baseline CD4 cell count at linkage to HIV care and at initiation of antiretroviral therapy on mortality in HIV-positive adult patients in Rwanda: a nationwide cohort study. *Lancet HIV* 2015;2:e376-384.

139. Asiimwe-Kateera B, Veldhuijzen N, Balinda JP *et al.* Combination Antiretroviral Therapy for HIV in Rwandan Adults: Clinical Outcomes and Impact on Reproductive Health up to 24 Months. *AIDS Res Treat* 2015;2015:740212.

140. Ondoa P, Gautam R, Rusine J *et al.* Twelve-Month Antiretroviral Therapy Suppresses Plasma and Genital Viral Loads but Fails to Alter Genital Levels of Cytokines, in a Cohort of HIV-Infected Rwandan Women. *PLoS One* 2015;10:e0127201.

141. Kamwesiga J, Mutabazi V, Kayumba J *et al.* Effect of selenium supplementation on CD4+ T-cell recovery, viral suppression and morbidity of HIV-infected patients in Rwanda: a randomized controlled trial. *AIDS* 2015;29:1045–52.

142. Mutimura E, Addison D, Anastos K *et al.* Trends in and correlates of CD4+ cell count at antiretroviral therapy initiation after changes in national ART guidelines in Rwanda. *AIDS* 2015;29:67–76.

143. Mpendo J, Mutua G, Nyombayire J *et al.* A Phase I Double Blind, Placebo-Controlled, Randomized Study of the Safety and Immunogenicity of Electroporated HIV DNA with or without Interleukin 12 in Prime-Boost Combinations with an Ad35 HIV Vaccine in Healthy HIV-Seronegative African Adults. *PLoS One* 2015;10:e0134287.

144. Gill MM, Hoffman HJ, Bobrow EA *et al.* Detectable Viral Load in Late Pregnancy among Women in the Rwanda Option B+ PMTCT Program: Enrollment Results from the Kabeho Study. *PLoS One* 2016;11:e0168671.

145. Smith SA, Burton SL, Kilembe W *et al.* Diversification in the HIV-1 Envelope Hyper-variable Domains V2, V4, and V5 and Higher Probability of Transmitted/Founder Envelope Glycosylation Favor the Development of Heterologous Neutralization Breadth. *PLoS Pathog* 2016;12.

146. Prentice HA, Lu H, Price MA *et al.* Dynamics and Correlates of CD8 T-Cell Counts in Africans with Primary Human Immunodeficiency Virus Type 1 Infection. *J Virol* 2016;90:10423–30.

147. Collins SE, Grant PM, Uwinkindi F *et al.* A Randomized Switch From Nevirapine-Based Antiretroviral Therapy to Single Tablet Rilpivirine/Emtricitabine/Tenofovir Disoproxil Fumarate in Virologically Suppressed Human Immunodeficiency Virus-1-Infected Rwandans. *Open Forum Infect Dis* 2016;3:ofw141.

148. Orikiiriza JT. Rollout of efavirenz-based regimens in option B+ in the prevention of mother-to-child transmission programs: challenges and lessons learned from a postexposure prophylaxis experience. *AIDS* 2016;30:N29-31.

149. Kayigamba FR, Franke MF, Bakker MI *et al.* Discordant Treatment Responses to Combination Antiretroviral Therapy in Rwanda: A Prospective Cohort Study. *PLoS One* 2016;11:e0159446.

150. Ndahimana J d’Amour, Riedel DJ, Mwumvaneza M *et al.* Drug resistance mutations after the first 12 months on antiretroviral therapy and determinants of virological failure in Rwanda. *Trop Med Int Health* 2016;21:928–35.

151. Ndahimana J d’Amour, Riedel DJ, Muhayimpundu R *et al.* HIV drug resistance mutations among patients failing second-line antiretroviral therapy in Rwanda. *Antivir Ther* 2016;21:253–9.

152. Mutagoma M, Ndahimana J d’Amour, Kayirangwa E *et al.* Prevalence of transmitted HIV-1 drug resistance among young adults attending HIV counselling and testing clinics in Kigali, Rwanda. *Antivir Ther* 2016;21:247–51.

153. Merci NM, Emerence U, Augustin N *et al.* CD4+ cells recovery in HIV positive patients with severe immunosuppression at HAART initiation at Centre Medico-Social Cor-Unum, Kigali. *Pan Afr Med J* 2017;26.

154. Nyombayire J, Anzala O, Gazzard B *et al.* First-in-Human Evaluation of the Safety and Immunogenicity of an Intranasally Administered Replication-Competent Sendai Virus-Vectored HIV Type 1 Gag Vaccine: Induction of Potent T-Cell or Antibody Responses in Prime-Boost Regimens. *J Infect Dis* 2017;215:95–104.

155. Rhee S-Y, Varghese V, Holmes SP *et al.* Mutational Correlates of Virological Failure in Individuals Receiving a WHO-Recommended Tenofovir-Containing First-Line Regimen: An International Collaboration. *EBioMedicine* 2017;18:225–35.

156. Mugwaneza P, Lyambabaje A, Umubyeyi A *et al.* Impact of maternal ART on mother-to-child transmission (MTCT) of HIV at six weeks postpartum in Rwanda. *BMC Public Health* 2018;18:1248.

157. Barouch DH, Tomaka FL, Wegmann F *et al.* Evaluation of a mosaic HIV-1 vaccine in a multicentre, randomised, double-blind, placebo-controlled, phase 1/2a clinical trial (APPROACH) and in rhesus monkeys (NHP 13-19). *The Lancet* 2018;392:232–43.

158. Woodson E, Goldberg A, Michelo C *et al.* HIV transmission in discordant couples in Africa in the context of antiretroviral therapy availability. *AIDS* 2018;32:1613–23.

159. Powers KA, Price MA, Karita E *et al.* Prediction of extended high viremia among newly HIV-1-infected persons in sub-Saharan Africa. *PLOS ONE* 2018;13:e0192785.

160. Tymejczyk O, Brazier E, Yiannoutsos C *et al.* HIV treatment eligibility expansion and timely antiretroviral treatment initiation following enrollment in HIV care: A metaregression analysis of programmatic data from 22 countries. *PLoS Med* 2018;15:e1002534.

161. Smith SA, Burton SL, Kilembe W *et al.* VH1-69 Utilizing Antibodies Are Capable of Mediating Non-neutralizing Fc-Mediated Effector Functions Against the Transmitted/Founder gp120. *Front Immunol* 2019;9.

162. Nsanzimana S, McArdle F, Remera E *et al.* Viral Suppression in a Nationwide Sample of HIV-Infected Children on Antiretroviral Therapy in Rwanda. *Pediatr Infect Dis J* 2019;38:149–51.

163. Mpendo J, Mutua G, Nanvubya A *et al.* Acceptability and tolerability of repeated intramuscular electroporation of Multi-antigenic HIV (HIVMAG) DNA vaccine among healthy African participants in a phase 1 randomized controlled trial. *PLoS One* 2020;15:e0233151.

164. Ruzagira E, Abaasa A, Karita E *et al.* Effect of seasonal variation on adult clinical laboratory parameters in Rwanda, Zambia, and Uganda: implications for HIV biomedical prevention trials. *PLoS One* 2014;9:e105089.

165. Mutwa PR, Fillekes Q, Malgaz M *et al.* Mid-dosing interval efavirenz plasma concentrations in HIV-1-infected children in Rwanda: treatment efficacy, tolerability, adherence, and the influence of CYP2B6 polymorphisms. *J Acquir Immune Defic Syndr* 2012;60:400–4.

166. Baden LR, Karita E, Mutua G *et al.* Assessment of the Safety and Immunogenicity of 2 Novel Vaccine Platforms for HIV-1 Prevention: A Randomized Trial. *Ann Intern Med* 2016;164:313–22.

167. Jaoko W, Karita E, Kayitenkore K *et al.* Safety and immunogenicity study of Multiclade HIV-1 adenoviral vector vaccine alone or as boost following a multiclade HIV-1 DNA vaccine in Africa. *PLoS One* 2010;5:e12873.

168. Ptak RG, Gallay PA, Jochmans D *et al.* Inhibition of human immunodeficiency virus type 1 replication in human cells by Debio-025, a novel cyclophilin binding agent. *Antimicrob Agents Chemother* 2008;52:1302–17.

169. Sundell J, Bienvenu E, Janzén D *et al.* Model-Based Assessment of Variability in Isoniazid Pharmacokinetics and Metabolism in Patients Co-Infected With Tuberculosis and HIV: Implications for a Novel Dosing Strategy. *Clin Pharmacol Ther* 2020;108:73–80.

170. Baden LR, Stieh DJ, Sarnecki M *et al.* Safety and immunogenicity of two heterologous HIV vaccine regimens in healthy, HIV-uninfected adults (TRAVERSE): a randomised, parallel-group, placebo-controlled, double-blind, phase 1/2a study. *Lancet HIV* 2020;7:e688–98.

171. Mutagoma M, Balisanga H, Malamba SS *et al.* Hepatitis B virus and HIV co-infection among pregnant women in Rwanda. *BMC Infectious Diseases* 2017;17:618.

172. Mutagoma M, Balisanga H, Sebuhoro D *et al.* Hepatitis C virus and HIV co-infection among pregnant women in Rwanda. *BMC Infect Dis* 2017;17:167.

173. Van de Perre P, Clumeck N, Steens M *et al.* Seroepidemiological study on sexually transmitted diseases and hepatitis B in African promiscuous heterosexuals in relation to HTLV-III infection. *Eur J Epidemiol* 1987;3:14–8.

174. Kestelyn P, Stevens AM, Bakkers E *et al.* Severe herpes zoster ophthalmicus in young African adults: a marker for HTLV-III seropositivity. *Br J Ophthalmol* 1987;71:806–9.

175. Van de Perre P, Bakkers E, Batungwanayo J *et al.* Herpes zoster in African patients: an early manifestation of HIV infection. *Scand J Infect Dis* 1988;20:277–82.

176. Mutagoma M, Nyirazinyoye L, Sebuhoro D *et al.* Syphilis and HIV prevalence and associated factors to their co-infection, hepatitis B and hepatitis C viruses prevalence among female sex workers in Rwanda. *BMC Infect Dis* 2017;17:525.

177. Drammeh B, Laperche S, Hilton J *et al.* Proficiency testing of viral marker screening in African blood centers: A multinational study. *Transfusion Clinique et Biologique* 2019;26:S99–100.

178. Mutwa PR, Boer KR, Rusine JB *et al.* Hepatitis B virus prevalence and vaccine response in HIV-infected children and adolescents on combination antiretroviral therapy in Kigali, Rwanda. *Pediatr Infect Dis J* 2013;32:246–51.

179. Rusine J, Ondoa P, Asiimwe-Kateera B *et al.* High Seroprevalence of HBV and HCV Infection in HIV-Infected Adults in Kigali, Rwanda. *PLoS One* 2013;8.

180. Veldhuijzen NJ, Dhont N, Vyankandondera J *et al.* Prevalence and concordance of HPV, HIV, and HSV-2 in heterosexual couples in Kigali, Rwanda. *Sex Transm Dis* 2012;39:128–35.

181. Nationwide community-based serological survey of HIV-1 and other human retrovirus infections in a central African country. Rwandan HIV Seroprevalence Study Group. *Lancet* 1989;1:941–3.

182. Sinayobye J d’Amour, Hoover DR, Shi Q *et al.* Prevalence of shingles and its association with PTSD among HIV-infected women in Rwanda. *BMJ Open* 2015;5:e005506.

183. Munyemana JB, Mukanoheli E, Nsabimana T *et al.* HCV Seroprevalence among HIV Patients and Associated Comorbidities at One Primary Health Facility in Rwanda. *Am J Trop Med Hyg* 2021;104:1747–50.

184. Murenzi G, Kim H-Y, Munyaneza A *et al.* Anogenital Human Papillomavirus and HIV Infection in Rwandan Men Who Have Sex With Men. *J Acquir Immune Defic Syndr* 2020;84:463–9.

185. Fischer W, Giorgi EE, Chakraborty S *et al.* HIV-1 and SARS-CoV-2: Patterns in the evolution of two pandemic pathogens. *Cell Host Microbe* 2021;29:1093–110.

186. Lepage P, Dabis F, Msellati P *et al.* Safety and immunogenicity of high-dose Edmonston-Zagreb measles vaccine in children with HIV-1 infection. A cohort study in Kigali, Rwanda. *Am J Dis Child* 1992;146:550–5.

187. Zhang X, Wallace OL, Domi A *et al.* Canine distemper virus neutralization activity is low in human serum and it is sensitive to an amino acid substitution in the hemagglutinin protein. *Virology* 2015;482:218–24.

188. Friedman-Klabanoff D, Ball A, Rutare S *et al.* Three Rwandan Children With Massive Splenomegaly and Epstein-Barr Virus-associated Lymphoproliferative Disorders: Case Presentations and the Literature Review. *J Pediatr Hematol Oncol* 2016;38:e158-161.

189. Clifford GM, Vaccarella S, Franceschi S *et al.* Comparison of Two Widely Used Human Papillomavirus Detection and Genotyping Methods, GP5+/6+-Based PCR Followed by Reverse Line Blot Hybridization and Multiplex Type-Specific E7-Based PCR. *J Clin Microbiol* 2016;54:2031–8.

190. Umulisa MC, Franceschi S, Baussano I *et al.* Evaluation of human-papillomavirus testing and visual inspection for cervical cancer screening in Rwanda. *BMC Womens Health* 2018;18:59.

191. Van Hecke E, Bugingo G. Prevalence of skin disease in Rwanda. *Int J Dermatol* 1980;19:526–9.

192. Centers for Disease Control (CDC). Human rabies--Rwanda. *MMWR Morb Mortal Wkly Rep* 1982;31:135.

193. Gascon J, Bruguera M, Corachan M *et al.* Serological survey of hepatitis B in northern Rwanda. *Trop Geogr Med* 1988;40:293–7.

194. Bogaerts J, Ricart CA, Van Dyck E *et al.* The etiology of genital ulceration in Rwanda. *Sex Transm Dis* 1989;16:123–6.

195. Mets T, Smitz J, Ngendahayo P *et al.* Hepatitis C virus infection in African patients with liver cirrhosis or primary hepatocellular carcinoma. *Scand J Gastroenterol* 1993;28:331–4.

196. Santiago ML, Bibollet-Ruche F, Gross-Camp N *et al.* Noninvasive detection of Simian immunodeficiency virus infection in a wild-living L’Hoest’s monkey (Cercopithecus Ihoesti). *AIDS Res Hum Retroviruses* 2003;19:1163–6.

197. Kateera F, Walker TD, Mutesa L *et al.* Hepatitis B and C seroprevalence among health care workers in a tertiary hospital in Rwanda. *Trans R Soc Trop Med Hyg* 2015;109:203–8.

198. Hoppe E, Pauly M, Gillespie TR *et al.* Multiple Cross-Species Transmission Events of Human Adenoviruses (HAdV) during Hominine Evolution. *Mol Biol Evol* 2015;32:2072–84.

199. McMorrow ML, Wemakoy EO, Tshilobo JK *et al.* Severe Acute Respiratory Illness Deaths in Sub-Saharan Africa and the Role of Influenza: A Case Series From 8 Countries. *J Infect Dis* 2015;212:853–60.

200. Smiley Evans T, Gilardi KVK, Barry PA *et al.* Detection of viruses using discarded plants from wild mountain gorillas and golden monkeys. *Am J Primatol* 2016;78:1222–34.

201. Ngabo F, Franceschi S, Baussano I *et al.* Human papillomavirus infection in Rwanda at the moment of implementation of a national HPV vaccination programme. *BMC Infect Dis* 2016;16:225.

202. Kalimba SE, Gahutu JB, Gatera M *et al.* Measles seroprevalence and outbreak in Rwanda: evidence from measles epidemiological surveillance and control. *International Journal of Infectious Diseases* 2014;21:146.

203. Madinda NF, Ehlers B, Wertheim JO *et al.* Assessing Host-Virus Codivergence for Close Relatives of Merkel Cell Polyomavirus Infecting African Great Apes. *Journal of Virology* 2016;90:8531–41.

204. Smiley Evans T, Lowenstine LJ, Gilardi KV *et al.* Mountain gorilla lymphocryptovirus has Epstein-Barr virus-like epidemiology and pathology in infants. *Sci Rep* 2017;7:5352.

205. Twagirumugabe T, Swaibu G, Walker TD *et al.* Hepatitis B virus strains from Rwandan blood donors are genetically similar and form one clade within subgenotype A1. *BMC Infectious Diseases* 2017;17:32.

206. Umutesi J, Simmons B, Makuza JD *et al.* Prevalence of hepatitis B and C infection in persons living with HIV enrolled in care in Rwanda. *BMC Infect Dis* 2017;17:315.

207. Twagirumugabe T, Swaibu G, Bergström T *et al.* Low prevalence of hepatitis C virus RNA in blood donors with anti-hepatitis C virus reactivity in Rwanda. *Transfusion* 2017;57:2420–32.

208. Umumararungu E, Ntaganda F, Kagira J *et al.* Prevalence of Hepatitis C Virus Infection and Its Risk Factors among Patients Attending Rwanda Military Hospital, Rwanda. *Biomed Res Int* 2017;2017:5841272.

209. Weldegebriel G, Mwenda JM, Chakauya J *et al.* Impact of rotavirus vaccine on rotavirus diarrhoea in countries of East and Southern Africa. *Vaccine* 2018;36:7124–30.

210. Operario DJ, Platts-Mills JA, Nadan S *et al.* Etiology of Severe Acute Watery Diarrhea in Children in the Global Rotavirus Surveillance Network Using Quantitative Polymerase Chain Reaction. *J Infect Dis* 2017;216:220–7.

211. Umuhoza T, Berkvens D, Gafarasi I *et al.* Seroprevalence of Rift Valley fever in cattle along the Akagera-Nyabarongo rivers, Rwanda. *J S Afr Vet Assoc* 2017;88:e1–5.

212. Andersson M, Kabayiza J-C, Elfving K *et al.* Coinfection with Enteric Pathogens in East African Children with Acute Gastroenteritis-Associations and Interpretations. *Am J Trop Med Hyg* 2018;98:1566–70.

213. Habyarimana T, Attaleb M, Mazarati JB *et al.* Detection of human papillomavirus DNA in tumors from Rwandese breast cancer patients. *Breast Cancer* 2018;25:127–33.

214. Mukanyangezi MF, Sengpiel V, Manzi O *et al.* Screening for human papillomavirus, cervical cytological abnormalities and associated risk factors in HIV-positive and HIV-negative women in Rwanda. *HIV Med* 2018;19:152–66.

215. Nyamusore J, Rukelibuga J, Mutagoma M *et al.* The national burden of influenza-associated severe acute respiratory illness hospitalization in Rwanda, 2012-2014. *Influenza Other Respir Viruses* 2018;12:38–45.

216. Sibomana H, Rugambwa C, Mwenda JM *et al.* Impact of routine rotavirus vaccination on all-cause and rotavirus hospitalizations during the first four years following vaccine introduction in Rwanda. *Vaccine* 2018;36:7135–41.

217. Seruyange E, Gahutu J-B, Muvunyi CM *et al.* Seroprevalence of Zika Virus and Rubella Virus IgG among blood donors in Rwanda and in Sweden. *Journal of Medical Virology* 2018;n/a.

218. Seruyange E, Ljungberg K, Muvunyi CM *et al.* Seroreactivity to Chikungunya and West Nile Viruses in Rwandan Blood Donors. *Vector Borne Zoonotic Dis* 2019;19:731–40.

219. Murthy S, O’Brien K, Agbor A *et al.* Cytomegalovirus distribution and evolution in hominines. *Virus Evol* 2019;5:vez015.

220. Makuza JD, Rwema JOT, Ntihabose CK *et al.* Prevalence of hepatitis B surface antigen (HBsAg) positivity and its associated factors in Rwanda. *BMC Infect Dis* 2019;19:381.

221. Norder H, Twagirumugabe T, Said J *et al.* High Frequency of Either Altered Pre-Core Start Codon or Weakened Kozak Sequence in the Core Promoter Region in Hepatitis B Virus A1 Strains from Rwanda. *Genes (Basel)* 2019;10.

222. Makuza JD, Liu CY, Ntihabose CK *et al.* Risk factors for viral hepatitis C infection in Rwanda: results from a nationwide screening program. *BMC Infect Dis* 2019;19:688.

223. Umutesi J, Liu CY, Penkunas MJ *et al.* Screening a nation for hepatitis C virus elimination: a cross-sectional study on prevalence of hepatitis C and associated risk factors in the Rwandan general population. *BMJ Open* 2019;9:e029743.

224. Twagirumugabe T, Saguti F, Habarurema S *et al.* Hepatitis A and E virus infections have different epidemiological patterns in Rwanda. *International Journal of Infectious Diseases* 2019;86:12–4.

225. Murenzi G. Preliminary Xpert® HPV testing results from a large study of women living with HIV in Rwanda. *J Virus Erad* 2019;5:6–7.

226. Mpunga T, Chantal Umulisa M, Tenet V *et al.* Human papillomavirus genotypes in cervical and other HPV-related anogenital cancer in Rwanda, according to HIV status. *Int J Cancer* 2020;146:1514–22.

227. Mukanyangezi MF, Rugwizangoga B, Manzi O *et al.* Persistence rate of cervical human papillomavirus infections and abnormal cytology in Rwanda. *HIV Med* 2019;20:485–95.

228. Markotter W, Geldenhuys M, Jansen van Vuren P *et al.* Paramyxo- and Coronaviruses in Rwandan Bats. *Trop Med Infect Dis* 2019;4.

229. Rugwizangoga B, Andersson ME, Kabayiza J-C *et al.* IFNL4 Genotypes Predict Clearance of RNA Viruses in Rwandan Children With Upper Respiratory Tract Infections. *Front Cell Infect Microbiol* 2019;9:340.

230. Nziza J, Goldstein T, Cranfield M *et al.* Coronaviruses Detected in Bats in Close Contact with Humans in Rwanda. *Ecohealth* 2020;17:152–9.

231. Lago BV, Mello FC, Kramvis A *et al.* Hepatitis B Virus Subgenotype A1: Evolutionary Relationships between Brazilian, African and Asian Isolates. *PLoS One* 2014;9.

232. Ozaras R, Inanc Balkan I, Yemisen M *et al.* Epidemiology of HBV subgenotypes D. *Clin Res Hepatol Gastroenterol* 2015;39:28–37.

233. Pourkarim MR, Lemey P, Amini-Bavil-Olyaee S *et al.* Novel hepatitis B virus subgenotype A6 in African-Belgian patients. *J Clin Virol* 2010;47:93–6.

234. Tagny CT, Diarra A, Yahaya R *et al.* Characteristics of blood donors and donated blood in sub-Saharan Francophone Africa. *Transfusion* 2009;49:1592–9.

235. Hübschen JM, Mugabo J, Peltier CA *et al.* Exceptional genetic variability of hepatitis B virus indicates that Rwanda is east of an emerging African genotype E/A1 divide. *J Med Virol* 2009;81:435–40.

236. Iradukunda PG, Habyarimana T, Niyonzima FN *et al.* Risk factors associated with hepatitis B and C in rural population of Burera district, Rwanda. *Pan Afr Med J* 2020;35.

237. Palacios G, Lowenstine LJ, Cranfield MR *et al.* Human Metapneumovirus Infection in Wild Mountain Gorillas, Rwanda. *Emerg Infect Dis* 2011;17:711–3.

238. Nyatanyi T, Nkunda R, Rukelibuga J *et al.* Influenza sentinel surveillance in Rwanda, 2008-2010. *J Infect Dis* 2012;206 Suppl 1:S74-79.

239. Spelman LH, Gilardi KVK, Lukasik-Braum M *et al.* Respiratory disease in mountain gorillas (Gorilla beringei beringei) in Rwanda, 1990-2010: outbreaks, clinical course, and medical management. *J Zoo Wildl Med* 2013;44:1027–35.

240. Kabayiza J-C, Andersson ME, Nilsson S *et al.* Real-time PCR identification of agents causing diarrhea in Rwandan children less than 5 years of age. *Pediatr Infect Dis J* 2014;33:1037–42.

241. Kabayiza J-C, Andersson ME, Nilsson S *et al.* Diarrhoeagenic microbes by real-time PCR in Rwandan children under 5 years of age with acute gastroenteritis. *Clin Microbiol Infect* 2014;20:O1128-1135.

242. Dutuze MF, Ingabire A, Gafarasi I *et al.* Identification of Bunyamwera and Possible Other Orthobunyavirus Infections and Disease in Cattle during a Rift Valley Fever Outbreak in Rwanda in 2018. *Am J Trop Med Hyg* 2020;103:183–9.

243. Li Y, Ndjango J-B, Learn GH *et al.* Eastern Chimpanzees, but Not Bonobos, Represent a Simian Immunodeficiency Virus Reservoir. *Journal of Virology* 2012;86:10776–91.

244. Wevers D, Metzger S, Babweteera F *et al.* Novel adenoviruses in wild primates: a high level of genetic diversity and evidence of zoonotic transmissions. *J Virol* 2011;85:10774–84.

245. Roy S, Vandenberghe LH, Kryazhimskiy S *et al.* Isolation and characterization of adenoviruses persistently shed from the gastrointestinal tract of non-human primates. *PLoS Pathog* 2009;5:e1000503.

246. Dutuze MF, Ingabire A, Gafarasi I *et al.* Identification of Bunyamwera and Possible Other Orthobunyavirus Infections and Disease in Cattle during a Rift Valley Fever Outbreak in Rwanda in 2018. *Am J Trop Med Hyg* 2020;103:183–9.

247. Li L, Kapoor A, Slikas B *et al.* Multiple diverse circoviruses infect farm animals and are commonly found in human and chimpanzee feces. *J Virol* 2010;84:1674–82.

248. Montecino-Latorre D, Goldstein T, Gilardi K *et al.* Reproduction of East-African bats may guide risk mitigation for coronavirus spillover. *One Health Outlook* 2020;2:2.

249. Anthony SJ, Johnson CK, Greig DJ *et al.* Global patterns in coronavirus diversity. *Virus Evol* 2017;3:vex012.

250. Mpunga T, Clifford GM, Morgan EA *et al.* Epstein-Barr virus prevalence among subtypes of malignant lymphoma in Rwanda, 2012 to 2018. *Int J Cancer* 2022;150:753–60.

251. Hall MD, Knowles NJ, Wadsworth J *et al.* Reconstructing geographical movements and host species transitions of foot-and-mouth disease virus serotype SAT 2. *mBio* 2013;4:e00591-00513.

252. Reeve R, Blignaut B, Esterhuysen JJ *et al.* Sequence-based prediction for vaccine strain selection and identification of antigenic variability in foot-and-mouth disease virus. *PLoS Comput Biol* 2010;6:e1001027.

253. Udahemuka JC, Aboge GO, Obiero GO *et al.* Risk factors for the incursion, spread and persistence of the foot and mouth disease virus in Eastern Rwanda. *BMC Vet Res* 2020;16:387.

254. Velkov S, Protzer U, Michler T. Global Occurrence of Clinically Relevant Hepatitis B Virus Variants as Found by Analysis of Publicly Available Sequencing Data. *Viruses* 2020;12.

255. Norder H, Twagirumugabe T, Said J *et al.* High Frequency of Either Altered Pre-Core StartCodon or Weakened Kozak Sequence in the CorePromoter Region in Hepatitis B Virus A1 Strainsfrom Rwanda. *Genes (Basel)* 2019;10.

256. Kostaki E-G, Karamitros T, Stefanou G *et al.* Unravelling the history of hepatitis B virus genotypes A and D infection using a full-genome phylogenetic and phylogeographic approach. *Elife* 2018;7.

257. Kamali I, Barnhart DA, Ndahimana J d’Amour *et al.* Prevalence and associated risk factors for hepatitis B and C viruses among refugee populations living in Mahama, Rwanda: A cross-sectional study. *PLoS One* 2021;16:e0257917.

258. Mpunga T, Chantal Umulisa M, Tenet V *et al.* Human papillomavirus genotypes in cervical and other HPV-related anogenital cancer in Rwanda, according to HIV status. *Int J Cancer* 2020;146:1514–22.

259. Chen Z, DeSalle R, Schiffman M *et al.* Niche adaptation and viral transmission of human papillomaviruses from archaic hominins to modern humans. *PLoS Pathog* 2018;14:e1007352.

260. Chen Z, Schiffman M, Herrero R *et al.* Evolution and taxonomic classification of alphapapillomavirus 7 complete genomes: HPV18, HPV39, HPV45, HPV59, HPV68 and HPV70. *PLoS One* 2013;8:e72565.

261. Hategeka C, Ogilvie G, Nisingizwe MP *et al.* Effect of human papilloma virus vaccination on sexual behaviours among adolescent women in Rwanda: a regression discontinuity study. *Health Policy Plan* 2020;35:1021–8.

262. Muhimpundu M-A, Ngabo F, Sayinzoga F *et al.* Screen, Notify, See, and Treat: Initial Results of Cervical Cancer Screening and Treatment in Rwanda. *JCO Glob Oncol* 2021;7:632–8.

263. Hirve S, Newman LP, Paget J *et al.* Influenza Seasonality in the Tropics and Subtropics - When to Vaccinate? *PLoS One* 2016;11:e0153003.

264. Lam TT, Tang JW, Lai FY *et al.* Comparative global epidemiology of influenza, respiratory syncytial and parainfluenza viruses, 2010-2015. *J Infect* 2019;79:373–82.

265. Zhong Q, Xu W, Wu Y *et al.* Patterns of synonymous codon usage on human metapneumovirus and its influencing factors. *J Biomed Biotechnol* 2012;2012:460837.

266. Shyaka A, Ugirabe MA, Wensman JJ. Serological Evidence of Exposure to Peste des Petits Ruminants in Small Ruminants in Rwanda. *Front Vet Sci* 2021;8:651978.

267. Dutuze MF, Mayton EH, Macaluso JD *et al.* Comparative characterization of the reassortant Orthobunyavirus Ngari with putative parental viruses, Bunyamwera and Batai: in vitro characterization and ex vivo stability. *J Gen Virol* 2021;102.

268. Rakau KG, Nyaga MM, Gededzha MP *et al.* Genetic characterization of G12P[6] and G12P[8] rotavirus strains collected in six African countries between 2010 and 2014. *BMC Infect Dis* 2021;21:107.

269. Ghosh A, Chattopadhyay S, Chawla-Sarkar M *et al.* In silico study of rotavirus VP7 surface accessible conserved regions for antiviral drug/vaccine design. *PLoS One* 2012;7:e40749.

270. Rasebotsa S, Mwangi PN, Mogotsi MT *et al.* Whole genome and in-silico analyses of G1P[8] rotavirus strains from pre- and post-vaccination periods in Rwanda. *Sci Rep* 2020;10:13460.

271. Rasebotsa S, Uwimana J, Mogotsi MT *et al.* Whole-Genome Analyses Identifies Multiple Reassortant Rotavirus Strains in Rwanda Post-Vaccine Introduction. *Viruses* 2021;13.

272. Ekpenyong ME, Edoho ME, Inyang UG *et al.* A hybrid computational framework for intelligent inter-continent SARS-CoV-2 sub-strains characterization and prediction. *Sci Rep* 2021;11:14558.

273. Yuan J, Wu Y, Jing W *et al.* Non-linear correlation between daily new cases of COVID-19 and meteorological factors in 127 countries. *Environ Res* 2021;193:110521.

274. Nelson CW, Ardern Z, Goldberg TL *et al.* Dynamically evolving novel overlapping gene as a factor in the SARS-CoV-2 pandemic. *Elife* 2020;9.

275. Mutesa L, Ndishimye P, Butera Y *et al.* A pooled testing strategy for identifying SARS-CoV-2 at low prevalence. *Nature* 2021;589:276–80.

276. Semakula M, Niragire F, Umutoni A *et al.* The secondary transmission pattern of COVID-19 based on contact tracing in Rwanda. *BMJ Glob Health* 2021;6.

277. Nsekuye O, Rwagasore E, Muhimpundu MA *et al.* Investigation of Four Clusters of Severe Acute Respiratory Syndrome Coronavirus 2 (SARS-CoV-2) in Rwanda, 2020. *Int J Environ Res Public Health* 2021;18.

278. Kremer C, Torneri A, Boesmans S *et al.* Quantifying superspreading for COVID-19 using Poisson mixture distributions. *Sci Rep* 2021;11:14107.

279. Butera Y, Mukantwari E, Artesi M *et al.* Genomic sequencing of SARS-CoV-2 in Rwanda reveals the importance of incoming travelers on lineage diversity. *Nat Commun* 2021;12:5705.

280. Liu W, Worobey M, Li Y *et al.* Molecular ecology and natural history of simian foamy virus infection in wild-living chimpanzees. *PLoS Pathog* 2008;4:e1000097.

281. Mazet JAK, Genovese BN, Harris LA *et al.* Human Respiratory Syncytial Virus Detected in Mountain Gorilla Respiratory Outbreaks. *Ecohealth* 2020;17:449–60.

282. Blinkova O, Victoria J, Li Y *et al.* Novel circular DNA viruses in stool samples of wild-living chimpanzees. *J Gen Virol* 2010;91:74–86.

283. Scherpenisse M, Schepp RM, Mollers M *et al.* Characteristics of HPV-specific antibody responses induced by infection and vaccination: cross-reactivity, neutralizing activity, avidity and IgG subclasses. *PLoS One* 2013;8:e74797.

284. Courtois G, Flack A, Jervis GA *et al.* Preliminary report on mass vaccination of man with live attenuated poliomyelitis virus in the Belgian Congo and Ruanda-Urundi. *Br Med J* 1958;2:187–90.

285. Vlietinck AJ, Van Hoof L, Totté J *et al.* Screening of hundred Rwandese medicinal plants for antimicrobial and antiviral properties. *J Ethnopharmacol* 1995;46:31–47.

286. Sindambiwe JB, Calomme M, Cos P *et al.* Screening of seven selected Rwandan medicinal plants for antimicrobial and antiviral activities. *J Ethnopharmacol* 1999;65:71–7.

287. Cos P, Hermans N, De Bruyne T *et al.* Further evaluation of Rwandan medicinal plant extracts for their antimicrobial and antiviral activities. *J Ethnopharmacol* 2002;79:155–63.

288. Riedel DJ, Taylor S, Simango R *et al.* Hepatitis C treatment outcomes using interferon- and ribavirin-based therapy in Kigali, Rwanda. *Trans R Soc Trop Med Hyg* 2016;110:495–7.

289. Franceschi S, Chantal Umulisa M, Tshomo U *et al.* Urine testing to monitor the impact of HPV vaccination in Bhutan and Rwanda. *Int J Cancer* 2016;139:518–26.

290. Je T, F N, P D *et al.* Effectiveness of Pentavalent Rotavirus Vaccine Under Conditions of Routine Use in Rwanda. *Clin Infect Dis* 2016;62 Suppl 2:S208-12.

291. Muvunyi CM, Harelimana JDD, Sebatunzi OR *et al.* Hepatitis B vaccination coverage among healthcare workers at a tertiary hospital in Rwanda. *BMC Research Notes* 2018;11:886.

292. Gupta N, Mbituyumuremyi A, Kabahizi J *et al.* Treatment of chronic hepatitis C virus infection in Rwanda with ledipasvir-sofosbuvir (SHARED): a single-arm trial. *Lancet Gastroenterol Hepatol* 2019;4:119–26.

293. Grant P, Shumbusho F, Nuil JIV *et al.* Safety and Efficacy of Limited Laboratory Monitoring for Hepatitis C Treatment: A Blinded Clinical Trial in Rwanda. *Hepatology Communications* 2020;4:569–76.

294. Binagwaho A, Wagner CM, Gatera M *et al.* Achieving high coverage in Rwanda’s national human papillomavirus vaccination programme. *Bull World Health Organ* 2012;90:623–8.

295. Abbink P, Kirilova M, Boyd M *et al.* Rapid Cloning of Novel Rhesus Adenoviral Vaccine Vectors. *J Virol* 2018;92.

296. Abbink P, Lemckert AAC, Ewald BA *et al.* Comparative seroprevalence and immunogenicity of six rare serotype recombinant adenovirus vaccine vectors from subgroups B and D. *J Virol* 2007;81:4654–63.

297. Mc Kenna P, Masyn S, Willems A *et al.* Leapfrogging with technology: introduction of a monitoring platform to support a large-scale Ebola vaccination program in Rwanda. *Hum Vaccin Immunother* 2021;17:3192–202.

298. DeBoer RJ, Shyirambere C, Driscoll CD *et al.* Treatment of Hodgkin Lymphoma With ABVD Chemotherapy in Rural Rwanda: A Model for Cancer Care Delivery Implementation. *JCO Glob Oncol* 2020;6:1093–102.

299. Jackson B, Harvey Y, Perez-Martin E *et al.* The selection of naturally stable candidate foot-and-mouth disease virus vaccine strains for East Africa. *Vaccine* 2021;39:5015–24.

300. Nsanzimana S, Penkunas MJ, Liu CY *et al.* Effectiveness of Direct-acting Antivirals for the Treatment of Chronic Hepatitis C in Rwanda: A Retrospective Study. *Clin Infect Dis* 2021;73:e3300–7.

301. Sayinzoga F, Umulisa MC, Sibomana H *et al.* Human papillomavirus vaccine coverage in Rwanda: A population-level analysis by birth cohort. *Vaccine* 2020;38:4001–5.

302. Verhaeghe T, Meulder MD, Hillewaert V *et al.* Capillary microsampling in clinical studies: opportunities and challenges in two case studies. *Bioanalysis* 2020;12:905–18.

303. Umereweneza D, Molel JT, Said J *et al.* Antiviral iridoid glycosides from Clerodendrum myricoides. *Fitoterapia* 2021;155:105055.
